# Air-Processed and Water-Stable Perovskite Solar Cells Enabled by a Fishing-Net-Inspired Interfacial Network

**DOI:** 10.1007/s40820-026-02295-5

**Published:** 2026-07-17

**Authors:** Muh Fadhil Albab, Muhammad Jahandar, Ah Ra Kim, Jinhee Heo, Yong Hyun Kim, Youngkyoo Kim, Gi-Hwan Kim, Ji-Youn Seo, Shinuk Cho, Soyeon Kim, Dong Chan Lim

**Affiliations:** 1 Energy and Environment Materials Research Division, Korea Institute of Materials Science (KIMS), Changwon, 51508 Republic of Korea; 2 Department of Smart Green Technology Engineering, Pukyong National University, Busan, 48513 Republic of Korea; 3 Materials Testing & Reliability Division, Korea Institute of Materials Science (KIMS), Changwon, 51508 Republic of Korea; 4 Organic Nanoelectronics Laboratory and KNU Institute for Nanophotonics Applications (KINPA), Department of Chemical Engineering, Kyungpook National University, Daegu, 41566 Republic of Korea; 5 Semiconductor Unit, Institute for Advanced Technology Convergence, Kyungpook National University, Daegu, 41566 Republic of Korea; 6 School of Materials Science and Engineering, Gyeongsang National University, Jinju, 52828 Republic of Korea; 7 Department of Nano Fusion Technology, Pusan National University, Busan, 46241 Republic of Korea; 8 Department of Semiconductor Physics & Engineering and Energy Harvest Storage Research Center, University of Ulsan, Ulsan, 44610 Republic of Korea

**Keywords:** Air-processed perovskite, Flexible perovskite solar cells, High efficiency, Interfacial modification, Water resistance

## Abstract

Practical deployment of perovskite solar cells is hindered by fragile interfaces that accelerate degradation under moisture, heat, ion migration, and mechanical stress, particularly during ambient processing. Here, we introduce a fishing-net-inspired interfacial molecular network that imparts intrinsic durability through coordination chemistry and interfacial dipole engineering. The metal-anchored hierarchical network integrates transition metal nodes, rigid small-molecule frameworks, and dense amine-functionalized polymer sub-networks into a netlike architecture that enhances charge extraction while suppressing bidirectional ion migration. Devices incorporating this interlayer achieve power conversion efficiencies of 26.19% (1.53 eV), 24.11% (1.61 eV), and 20.00% (1.77 eV), with open-circuit voltages and fill factors all exceeding 90% of the Shockley–Queisser radiative limit. Notably, this performance is maintained even in wide-bandgap flexible devices. Flexible perovskite solar cells fabricated entirely under ambient air achieve 23.03% efficiency and retain 95% of their initial performance after 10,000 bending cycles. Moreover, the devices exhibit suppressed degradation during direct water immersion and reach a *T95* exceeding 2000 h under ambient conditions without encapsulation, establishing a broadly applicable interfacial design strategy for durable optoelectronics.

**Supplementary Information:**

The online version contains supplementary material available at 10.1007/s40820-026-02295-5.

## Highlights

- A metal-anchored molecular net interlayer strategy enables highly efficient inverted perovskite solar cells by optimizing interfacial energetics and charge transport.
- The metal–amine coordinated network provides a universal interfacial platform, regardless of bandgap or substrate, to suppress ion migration and prevent moisture-induced degradation.
- The optimized devices deliver high efficiency together with excellent durability, maintaining >95% of initial performance after 2000 h storage, >95% retained performance after 10,000 bending cycles, and slow degradation after water exposure

**Supplementary Information:**

The online version contains supplementary material available at 10.1007/s40820-026-02295-5.

## Graphical abstract

**Figure:**
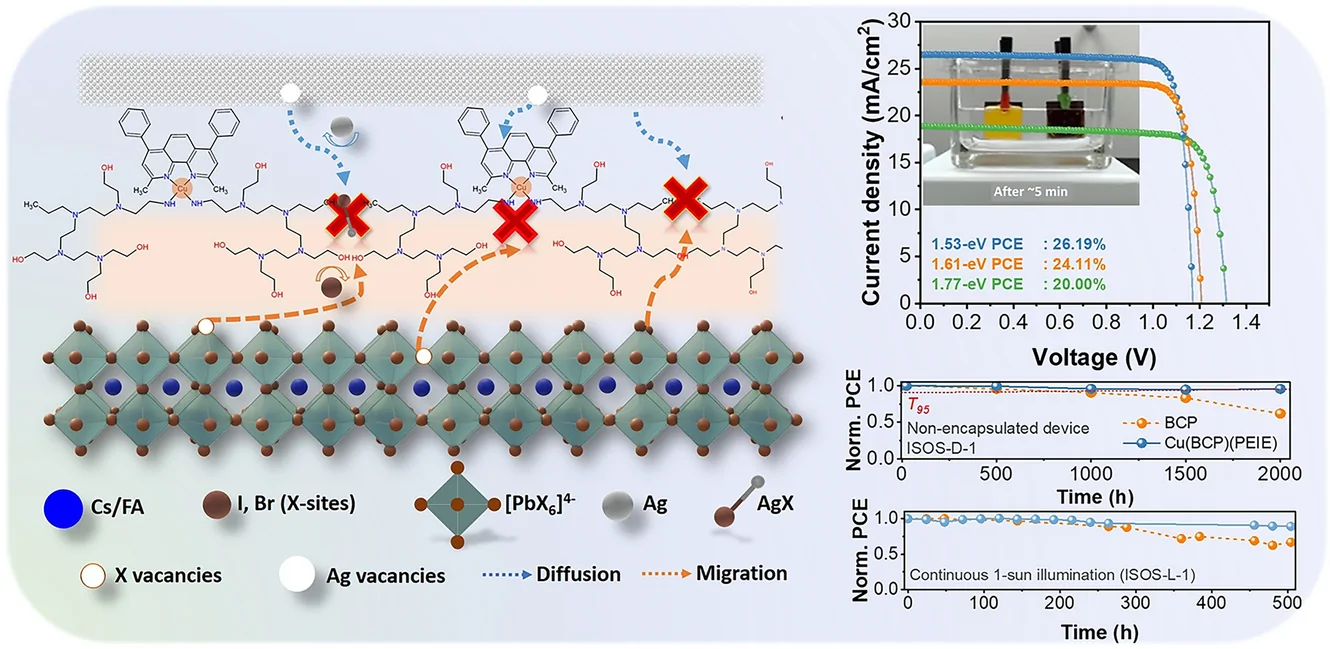

## Introduction

Halide perovskite solar cells (PSCs) have transformed photovoltaics, achieving power conversion efficiencies (PCEs) exceeding 26%, rivaling crystalline silicon [1–3]. However, commercialization remains limited by poor stability, particularly humidity-induced interfacial degradation, one of the most practically limiting yet under-addressed failure mechanisms, especially under ambient air conditions essential for scalable manufacturing [4–8]. The electron transporting layer (ETL)/cathode interface is particularly critical, governing charge extraction, recombination losses, and long-term contact stability. Effective interfacial layers must simultaneously enable efficient electron transport, suppress non-radiative recombination, and provide intrinsic resistance to moisture and oxygen ingress, requirements that remain challenging to achieve simultaneously within a unified material design [8–10].

Conventional cathode interlayers—ranging from inorganic materials like SnO_2_ [11] or bismuth [12], phenanthroline-based small molecules (e.g., bathocuproine (BCP)) [13, 14], single- or double-layer structures [15], to surface treatments using dipole-controlled polymers—have addressed subsets of these requirements. However, these approaches share a fundamental limitation of predominantly targeting individual degradation mechanisms and offer little intrinsic resistance to humidity-induced interfacial failure. BCP-based interlayers are susceptible to crystallization and delamination under humidity, dipole-engineered polymers lack structural robustness under continuous moisture exposure, and inorganic interlayers are incompatible with ambient processing and flexible substrates. Although Cu^2^⁺-based coordination chemistry has shown promise on the hole transporting layer side of n-i-p devices [16], its application at the ETL/cathode interface of p-i-n architectures remains largely unexplored. These shortcomings are most acute under air-processed conditions and on flexible substrates, representing a critical unresolved gap for scalable manufacturing.

State-of-the-art stability demonstrations consequently rely on extrinsic encapsulation strategies, including epoxy sealants or inorganic barrier layers [17, 18], to meet ISOS standard testing protocols [19]. Once encapsulation integrity is compromised, however, device degradation typically accelerates rapidly, exposing the absence of genuine material-level interfacial protection. This limitation is particularly severe for flexible and air-processed PSCs, which are essential for scalable manufacturing but are inherently more vulnerable to environmental stress [20–22]. The absence of intrinsic, humidity-resistant interfacial designs that remain compatible with ambient air processing thus represents a critical and unresolved gap in the field.

Beyond conventional solar harvesting, PSCs hold immense promise as ubiquitous power sources for the Internet of Things (IoT) sensors under low light. More ambitiously, PSCs could power sensors in smart agriculture and aquaculture, monitoring parameters like water temperature, ion concentration, or nutrient levels in real time [23, 24]. However, such emerging applications impose much stricter demands on stability—requiring operation under water immersion, high salinity, and fluctuating thermal conditions [25]. To date, only a handful of studies have tackled these extremes, often employing passivation techniques such as organic ammonium salts or polymeric coatings to stabilize the perovskite surface [26]. Fundamental material-level innovations that intrinsically enhance humidity and moisture stability while preserving high efficiency remain scarce, particularly in air-processed, flexible devices suitable for scalable manufacturing.

Here, we introduce a metal-anchored molecular inter-net concept, inspired by the architecture of a fishing net, that integrates coordination chemistry and interfacial dipole engineering to directly address humidity-induced interfacial degradation in perovskite devices fabricated under ambient air conditions. In a representative ternary implementation, Cu^2^⁺ ions act as anchoring nodes, bathocuproine (BCP) forms rigid molecular frameworks, and amine-rich polymers create dense sub-networks, collectively suppressing bidirectional ion migration and oxidative degradation while enhancing charge extraction and hydrophobicity. This strategy is generalizable to other transition metal anchors and amine-functionalized polymers. Perovskite solar cells with a 1.61 eV bandgap achieve power conversion efficiencies exceeding 24% (certified 24.07%), among the highest reported to date. Across bandgaps of 1.53 and 1.77 eV, the devices reach efficiencies of 26.19% (certified 25.18%) and 20.00% (certified 19.19%), respectively, with open-circuit voltages and fill factors exceeding 90% of the detailed Shockley–Queisser limit. The devices retain over 95% of their initial efficiency after 2000 h under the ISOS-D-1 protocol and exhibit exceptional unencapsulated stability during direct water immersion. Crucially, the interfacial network remains effective under manufacturing-relevant conditions. Completely air-processed flexible PSCs achieve a PCE of 23.03%, preserve over 95% of their initial efficiency after 10,000 bending cycles. The results establish the fishing-net-inspired molecular architecture as a general design principle or simultaneously achieving high efficiency and intrinsic durability against humidity-induced degradation in perovskite optoelectronics.

## Experimental Section

### Materials

Solvents: N,N-dimethylformamide (DMF, Sigma-Aldrich, 99.8%), dimethylsulfoxide (DMSO, Sigma-Aldrich, 99.8%), isopropanol (IPA, General-Reagent, 99.7%), ethanol (General-Reagent, 99.5%), chlorobenzene (CB, Sigma-Aldrich, 99.8%). Ethyl acetate (Sigma-Aldrich, 99.8%).

Reagents: Formamidinium iodide (FAI, Great-Cell, 99.9%), cesium bromide (CsBr, AnhydroBeads, 99.999%, Sigma-Aldrich), lead(II) iodide (PbI_2_, Sigma-Aldrich), lead(II) bromide (PbBr_2_, Sigma-Aldrich), cesium iodide (CsI, AnhydroBeads, 99.999%, Sigma-Aldrich), [6, 6]-phenyl-C61-butyric acid methyl ester (PC_61_BM, Nano-C), bathocuproine (BCP, Sigma-Aldrich), [2-(3,6-dimethoxy-9H-carbazol-9-yl)ethyl]phosphonic acid (MeO-2PACz, TCI, 98.0%), [4-(3,6-dimethoxy-9H-carbazol-9-yl)butyl]phosphonic acid (MeO-4PACz, TCI, 98.0%), [4-(7 H -dibenzo[c,g]carbazol-7-yl)butyl]phosphonic acid (4PADCB, TCI, > 98%) phenethylammonium iodide (PEAI, Sigma-Aldrich). Polyethylenimine ethoxylated (PEIE, Sigma-Aldrich). Copper(II) chloride (CuCl_2_, Sigma-Aldrich), 2-phenetylammonium chloride (PEACl, Sigma-Aldrich).

### Preparation of NiOx Nanoparticles, Metal Complexes, and Perovskite Precursor Solution

#### Preparations of the NiOx Nanoparticles

Nickel nitrate hexahydrate (Ni(NO_3_)_2_·6H_2_O) and sodium hydroxide (NaOH) were used as received without further purification. Ni(NO_3_)_2_·6H_2_O (1.85 mg) was dissolved in deionized water, and a 10 M aqueous NaOH solution was added dropwise until the pH reached approximately 10. The resulting mixture was stirred at room temperature for 1 h, during which nickel hydroxide (Ni(OH)_2_) was formed in situ via a chemical co-precipitation reaction between aqueous nickel nitrate and the basic solution. The Ni(OH)_2_ precipitate was subsequently collected, thoroughly washed, and freeze-dried for 24 h. The dried powder was then calcined at 270 °C for 2 h to obtain black powder NiO_x_ nanoparticles.

#### Metal Complex Preparation

0.5 mg mL^−1^ (1.40 mM) BCP was dissolved in IPA and stirred overnight at RT to completely dissolve BCP, namely solution A. Separately, 5 mg of CuCl_2_ (1.86 mM) was dissolved in 20 mL of IPA and stirred at RT for 15 min using a magnetic stirrer (500 rpm) until fully dissolved. Next, the solute CuCl_2_ (solution B) was filtered through a 0.22 *µ*m PTFE syringe filter to remove any particulate impurities. The CuCl_2_ solution (5 *µ*L) was added dropwise to the 20 mL of 0.5 mg mL^−1^ BCP solution (molar ratio 1:149) under continuous stirring at 40 °C. The mixture was stirred at 40 °C for 2 h to allow Cu^2^⁺ to coordinate with the nitrogen atoms of BCP’s phenanthroline backbone, forming the [Cu(BCP)]^2^⁺ intermediate. To form Cu(BCP)(PEIE), 0.025 wt% of PEIE solution was added to the mixed solution. The mixture was stirred at 40 °C for 4 h to allow the amine groups of PEIE to coordinate with the remaining coordination sites of Cu^2^⁺.

#### 1.53-eV Perovskite Precursor Solutions

The FA_0.95_Cs_0.05_PbI_3_ precursor solution was synthesized by dissolving a mixture comprising 18.2 mg of CsI, 228.4 mg of FAI, and 661.5 mg of PbI_2_ in a solvent mixture consisting of 800 *µ*L of DMF and 200 *µ*L of DMSO, resulting in a 1.4 M solution with a 2.5% molar excess of PbI_2_ relative to the stoichiometric ratio. The solutions were stirred at 60 °C for 2 h and filtered using a 0.45-*µ*m polytetrafluoroethylene membrane before use.

#### 1.61-eV Perovskite Precursor Solutions

The FA_0.88_Cs_0.12_PbI_2.64_Br_0.36_ perovskite precursor solution was comprised of 301 mg of FAI, 53.2 mg of CsBr, 806.8 mg of PbI_2_, 91.8 mg of PbBr_2_ in 1050 *µ*L DMF, and 150 *µ*L DMSO. The solutions were stirred at 60 °C for 2 h and filtered using a 0.45-*µ*m polytetrafluoroethylene membrane before use.

#### 1.77-eV Perovskite Precursor Solutions

The 1.4 M FA_0.8_Cs_0.2_Pb(I_0.6_Br_0.4_)_3_ WBG perovskite precursors were prepared by dissolving 72.74 mg of CsI, 192.64 mg of formamidine hydroiodide (FAI), 268.15 mg of PbI_2_, and 308.28 mg of PbBr_2_ in 1 mL of a mixed solvent of DMF and DMSO in a 4:1 volume ratio. The solutions were stirred at 40 °C for 2 h and filtered using a 0.45-*µ*m polytetrafluoroethylene membrane before use.

### Device Fabrication

#### Fabrication of Rigid PSCs

ITO-patterned substrates were first sonicated in a solution containing acetone and IPA for 10 min each before being dried at 100 °C for one hour. After extensive cleaning, ITO substrates underwent a 1000 s UV–ozone treatment. 4PADCB (0.5 mg mL^−1^) was dissolved in anhydrous ethanol and deposited by spin-coating at 3000 revolutions per minute (rpm) for 30 s, followed by annealing at 100 °C for 10 min. The perovskite layer was spin-coated at 1000 rpm for 3 s and then at 4000 rpm for 20 s. At 8 s before the end of the program, 200 *µ*L of ethyl acetate was quickly dropped onto the substrate, and then, the films were annealed at 100 °C for 20 min. For the 1.53 eV FA_0.95_Cs_0.05_PbI_3_ perovskite, the coating recipe was 1000 rpm for 10 s and the second step at 4000 rpm for 40 s. During the spin-coating process, 100 *µ*L of CB antisolvent was dripped onto the wetted film 10 s before the end of the process and then annealed at 100 °C for 30 min. For the wide-bandgap 1.77 eV perovskite, the spin speed was 1000 rpm for 3 s and the second step at 5000 rpm for 40 s. During the spin-coating process, 100 *µ*L of anisole antisolvent was dripped onto the wetted film 5 s before the end of the process and then annealed at 100 °C for 15 min. Afterward, 50 *µ*L of PEAI solution (3 mg mL^−1^ in IPA) was spin-coated (5000 rpm) on top of the top perovskite film and annealed for 2 min. The electron transport layer of PCBM (20 mg mL^−1^ in CB) was spin-coated at 2000 rpm for 30 s and annealed at 100 °C for 10 min. The device was cooled down for 5 min. The BCP (0.5 mg mL^−1^ in IPA) or Cu(BCP)(PEIE) solution was subsequently spin-coated at 4000 rpm for 20 s and annealed at 100 °C for 5 min. Finally, a 100-nm-thick Ag electrode was deposited by thermal evaporation (< 4 × 10^−6^ Torr).

#### Fabrication of Flexible PSCs

For the flexible device, the complete architecture is PET(ITO)/NiO_x_ nanoparticles/MeO-4PACz/perovskite/PEAI/PCBM/buffer layer/Ag. The PET/ITO substrates were pre-cleaned in subsequent deionized water (DIW), acetone, and IPA for 10 min, respectively. Next, the substrates were dried at 100 °C and treated with UV–ozone for 10 min, and NiOx nanoparticle solution (10 mg mL^−1^) in mixed DIW:IPA (9:1 v/v) was spin-coated at 3000 rpm for 30 s, followed by thermal annealing at 100 °C for 10 min. Afterward, MeO-4PACz solution was spin-coated at 3000 rpm for 30 s, followed by annealing at 100 °C for 10 min. The perovskite film coating’s recipe was the same for both flexible and rigid substrates. After perovskite film coating, PCBM was dynamically spin-coated, annealed at 100 °C for 10 min, followed by buffer layer coating. Finally, the 100 nm Ag electrode was thermally evaporated on top of the buffer layer.

#### Fabrication of Air-Processed Flexible and Rigid PSCs

For the air-processed perovskite, the device architecture is ITO/NiOx nanoparticles/Me-4PACz/perovskite/PEACl/PCBM/BCP or modified BCP/Ag for both flexible and rigid substrates. The PEACl passivation layer was selected based on our previous reported study [27]. All solution-processed steps (NiO_x_, Me-4PACz, perovskite, PCBM, buffer layer) was performed in ambient air (outside glovebox) at 18–25 °C and 30–50% RH. The spin-coating recipe for all layers was same for those devices fabricated in inert control environment.

### Device Analysis

The current density–voltage (*J*–*V*) curves were characterized in ambient air using a Keithley 2400 A source with SimuLight PLS300 under AM 1.5 G simulated illumination of 100 Mw cm^**−**2^. The light intensity at each wavelength was calibrated using a standard monocrystalline Si photovoltaic cell (K801S-K073). Device measurements were taken at a scan speed of 100 mV/s. The illumination area of the devices was 0.048 cm^2^, defined by a shadow mask coated with black paint. All characterizations were performed in ambient air with a relative humidity of approximately 30–50%. For indoor photovoltaic device performance measurements, a warm white LED lamp (2700 K) was utilized. The stabilized output of the devices was achieved by measuring the power output of the illuminated device at a constant current close to the maximum power point derived from the *J*–*V* curve. The active area for the indoor light measurement was similar to that of a 1 sun illumination *J*–*V* sweep. The air storage stability was measured periodically at the relative humidity of 20–45% and temperature ranging from 20–30 °C. For photostability evaluation, the devices underwent continuous light exposure at 100 mW cm^−2^ (equivalent to 1 sun) using a Solar Cell Reliability Test System (K3600, McScience). The *J*–*V* characteristics of each device were measured at designated intervals to track performance throughout the exposure duration. To investigate the charge recombination mechanism, photo-CELIV curves were measured at different light intensities. The Pb and Cs leakage after water immersion was measured using an inductively coupled plasma–mass spectrometer (ICP–MS, Agilent 7850). The optical absorbance and transmittance spectra were measured using a UV–Vis spectrophotometer (Varian, Cary5000). The surface-wetting behavior was measured using an SEO Phoenix-300 contact angle analyzer. The surface morphology of films was observed by atomic force microscopy (AFM) (Park, NX10 AFM). The field-emission scanning electron micrographs were obtained with a JIB-4601F (JEOL). The scanning transmission electron microscopy (STEM) was conducted at 200 kV using JEM-ARM 200F equipment, and the sample preparations were performed on a Helios 5 CX dual-beam microscope equipped with a cryogenic system. The sample for TEM measurement was prepared after 48 h of thermal treatment at 100 °C. The time-of-flight secondary ion mass spectrometry (ToF–SIMS) for compositional depth profiling of perovskite solar cells was carried out using ToF–SIMS M6 system from IONTOF, operated in the spectral mode using LMIG, 30 keV Bi^3+^ ion beam with ion current 0.55 pA. For depth profiling, a 2-keV Cs^+^ sputter beam with a current of 50 nA was used to remove material layer by layer in interlaced mode. Ultraviolet photoelectron spectroscopy (UPS) with a He I source (21.2 eV) was used to investigate the work functions of the device with different buffer layers. The EIS measurement was taken with an AC impedance analyzer Compactstat electrochemical interface (IVIUM Technologies) in the frequency range from 100 to 1 MHz, with a perturbation amplitude of 0.1 V and a bias voltage close to *Voc* under AM dark conditions.

## Results and Discussion

### Chemical Design of Mixed-Ligand Complexes

**Fig. 1:**
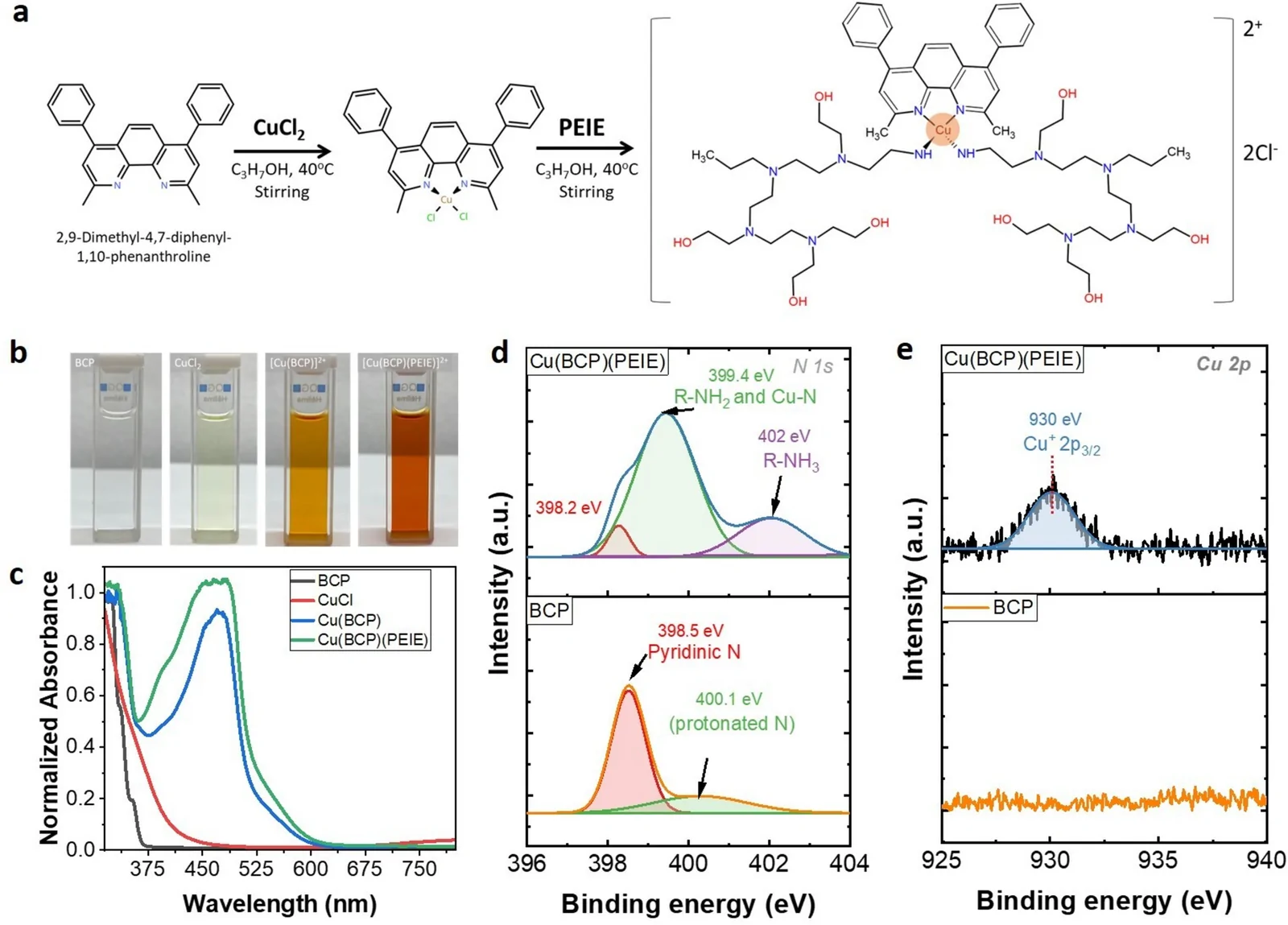
Proposed reaction mechanism of mixed-ligand complexes. **a** Cu(BCP)(PEIE) complexation. **b** Photographs of precursor solutions (BCP, CuCl_2_, Cu(BCP)^2+^, Cu(BCP)(PEIE)) upon complex formation. **c** Absorbance profile of different solutions. **d** XPS N 1*s* spectra of BCP and Cu(BCP)(PEIE). **e** XPS Cu 2*p* spectrum of Cu(BCP)(PEIE) confirming the presence of Cu element

Owing to their vacant d-orbitals, Cu^2^⁺ ions exhibit strong coordination tendencies with donor atoms, enabling the formation of stable complexes through interactions with nitrogen, oxygen, or sulfur sites [28]. In this system, Cu^2^⁺ coordinates preferentially with the nitrogen functionalities of BCP and PEIE (polyethylenimine ethoxylated), while PEIE further contributes amino and ethoxyl groups, thereby reinforcing the coordination network and enhancing the chemical robustness and interfacial integrity of the modified layer. Upon the addition of CuCl_2_ to BCP, Cu^2^⁺ coordinates to the pyridine-type nitrogen atoms of BCP, yielding Cu(BCP)^2+^ complexes. Subsequent introduction of PEIE results in strong chelation of Cu^2^⁺ by its primary and secondary amine groups, forming a ternary Cu(BCP)(PEIE) complex (Fig. 1a). The mixed-ligand environment stabilizes the Cu coordination center and enables synergistic modulation of the complex electronic structure. The formation of such complexes is visually evident in the solution color changes (Fig. 1b). Solutions of pristine BCP and CuCl_2_ are colorless and pale yellow, respectively. The Cu(BCP)^2+^ complex exhibits an orange hue, which intensifies further upon the addition of PEIE to form Cu(BCP)(PEIE). These color transitions reflect new electronic interactions, consistent with ligand-to-metal charge transfer commonly observed in Cu^2^⁺ coordination complexes [29, 30]. UV–Vis absorption spectra further support this coordination process (Fig. 1c). While pristine BCP displays its characteristic π–π* transitions (peaks at around 340–350 nm), the Cu(BCP)^2+^ and Cu(BCP)(PEIE) solutions show additional absorption shoulders in the visible region (470 nm), which can be attributed to Cu–N charge transfer interactions [31, 32]. The slight redshift and increased intensity observed in Cu(BCP)(PEIE) relative to Cu(BCP) imply stronger ligand field stabilization due to the synergistic chelation effect of the metal complex with amine-containing polymers, e.g., polyethylenimine [33].

X-ray photoelectron spectroscopy (XPS) provides more direct evidence for the coordination mechanism. Figure 1d exhibits the N 1*s* core-level spectrum of pristine BCP (bottom), which reveals peaks at 398.5 and 400.1 eV, assigned to pyridinic N and a weakly coordinated/protonated pyridinic N in the phenanthroline environment, showing a broadened shoulder due to surface adsorption of trace moisture or the inherent asymmetry of the pyridinic N peak. After complexation, the N 1*s* spectrum of Cu(BCP)(PEIE) exhibits a new feature at 399.4 eV, which can be ascribed to Cu–N coordination and partially protonated amines (R–NH_2_···Cu). Additionally, a higher binding energy component at 402 eV corresponds to R–NH_3_^+^ species, suggesting that some amine groups of PEIE are protonated during the coordination process. These findings are consistent with previous studies where transition metal ions coordinate with multidentate amines, shifting the N 1*s* core level due to electron withdrawal by the Cu^2+^ center [34, 35]. The Cu 2*p*_3/2_ core-level peak (Fig. 1e) observed at ~ 930 eV is consistent with Cu in the divalent oxidation state, in agreement with literature values for Cu^2⁺^ in nitrogen-coordinated complexes [36]. The reduced satellite intensity relative to uncoordinated CuCl_2_ is attributed to strong covalent back-donation from the BCP and PEIE nitrogen donors into the Cu d-orbital manifold, which partially quenches the ligand-to-metal charge transfer transitions responsible for shake-up excitations. This coordination-induced satellite suppression reflects the strength of the Cu^2⁺^–ligand interaction within the mixed-ligand chelate network and indicates the formation of a Cu(BCP)(PEIE) chelate, where BCP and PEIE function as complementary ligands. This cooperative binding is anticipated to suppress interfacial defect states, improve energy-level alignment, and strengthen the chemical stability of the electron transport interface in inverted perovskite solar cells.

### Suppression of Ions/Ag Migration

**Fig. 2:**
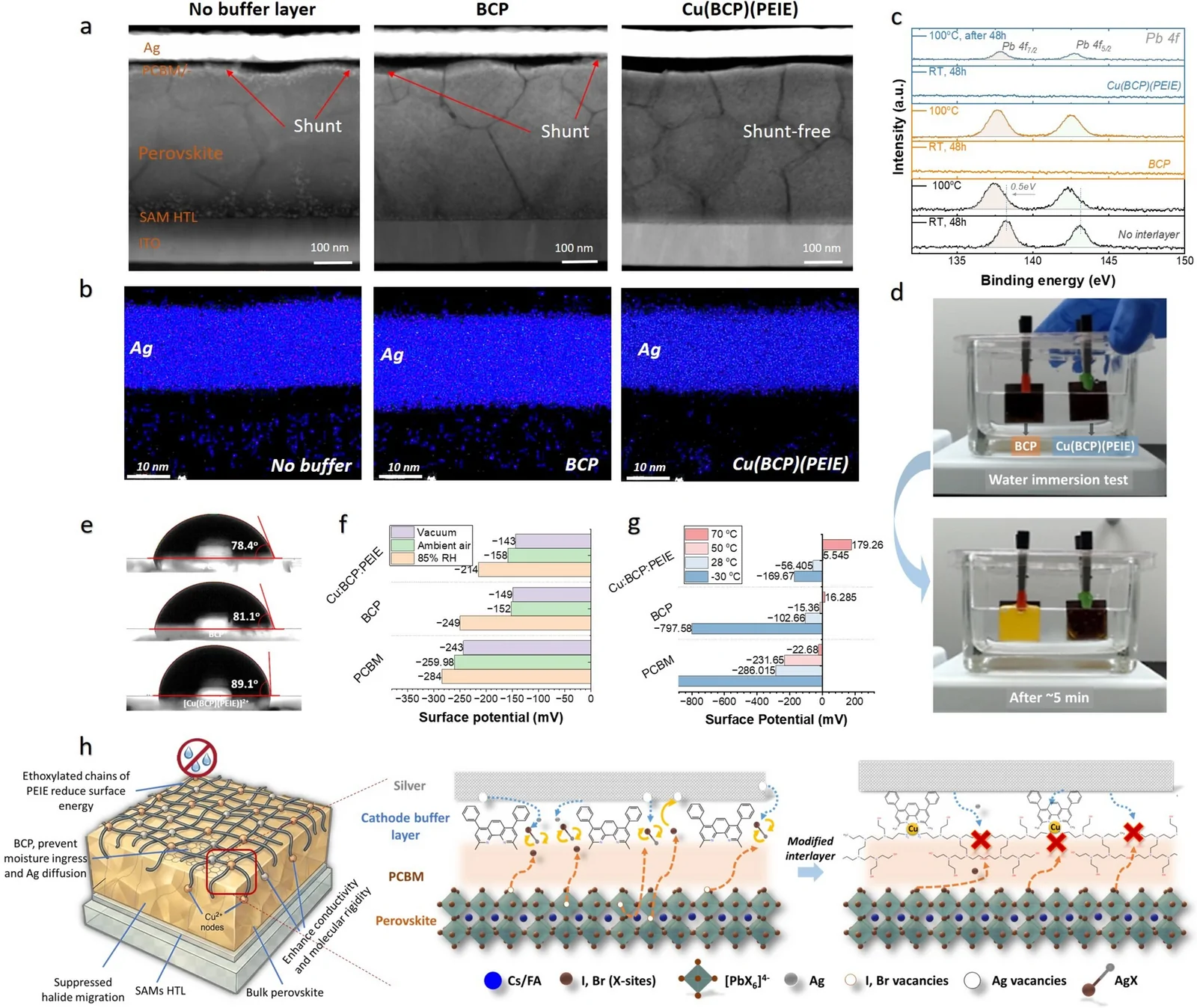
Characterization of film surface and interfaces. **a** Cross-sectional HR-TEM images of perovskite solar cells with different buffer layers after 48 h of thermal aging at 100 °C in ambient air. **b** HAADF-STEM images and the EDX maps of the Ag element. **c** XPS Pb 4*f* spectra of Perovskite/PCBM/with or without buffer layer/5 nm Ag, before and after thermal degradation test. **d** Water immersion test of perovskite solar cells without a top Ag electrode. **e** Static water contact angle of perovskite/PCBM with different buffer layers. **f** Surface potential under three environmental conditions (vacuum, ambient air, 85% RH). **g** Surface potential measured after thermal stress at a series of temperatures (− 30 to 70 °C). **h** A fishing-net-inspired design principle, in which weighted nodes, rigid meshes, and fine sub-networks are integrated to form a hierarchical molecular network. Inset: a schematic illustration of silver diffusion and halide migration in BCP and Cu(BCP)(PEIE)

One of the major obstacles to achieving highly stable PSCs lies in controlling interfacial degradation processes such as halide migration, metal diffusion, and perovskite decomposition. To directly demonstrate the mitigation of these effects, we systematically investigated the role of buffer layers in inverted PSCs (Fig. S1). High-resolution cross-sectional transmission electron microscopy (HR-TEM) images of thermally aged samples (Fig. 2a) reveal noticeable differences in ETL interfacial morphology. Devices without a buffer layer exhibit severe shunting pathways penetrating from the Ag electrode into the perovskite, which can act as fast channels for ion and electron leakage. Although the introduction of BCP reduces direct contact between Ag and the perovskite, shunting features are still visible, indicating incomplete suppression of interfacial degradation. In sharp contrast, the Cu(BCP)(PEIE)-modified devices present a well-defined, continuous, and shunt-free interface, suggesting that the hybrid interlayer effectively blocks the formation of electrically detrimental pathways and ion migration. Moreover, as shown in Fig. 2b, the high-angle annular dark-field scanning transmission electron microscopy (HAADF-STEM) coupled with energy-dispersive X-ray spectroscopy (EDX) mapping of Ag after thermal aging (100 °C, 48 h in ambient air) reveals pronounced interfacial degradation in the absence of an interlayer, with Ag penetrating deeply into the perovskite absorber. In BCP-buffered devices, an edge-thickened Ag electrode is observed, indicating downward Ag diffusion toward the perovskite, accompanied by substantial redistribution of iodide species from the perovskite layer (Fig. S2). This bidirectional elemental intermixing reflects severe interfacial ion migration under combined thermal and moisture stress. By contrast, devices incorporating the Cu(BCP)(PEIE) interlayer exhibit effectively suppressed Ag diffusion and halide migration, maintaining abrupt interfaces and spatially uniform elemental distributions. To further substantiate these findings, TOF–SIMS depth profiling was performed to provide quantitative, layer-resolved analysis of ion migration across the device stack. As depicted in Fig. S3, the depth profiles of the BCP-based aged device reveal that I⁻ secondary ion signals extend continuously throughout the entire layer sequence, confirming that thermal aging drives pervasive cross-layer ion migration from the perovskite absorber into metallic electrode. In the Cu(BCP)(PEIE)-based device, the I⁻ profile shows a steep gradient that terminates at the Cu(BCP)(PEIE)/PCBM interface, demonstrating that the interlayer acts as an effective physicochemical barrier against halide migration.

The devices were further subjected to XPS analysis after degradation test at ambient air (RH ~ 30%) for 48 h at RT and 100 ℃, as shown in Fig. 2c. For the no-buffer-layer devices, the binding energy downshifts by ~ 0.5 eV relative to the pristine sample. This negative binding energy shift suggests the formation of under-coordinated Pb after thermal stress following halide loss and subsequent electronic redistribution. The concurrent increase in integrated Pb 4*f* area (Table S1) reflects the accumulation of these under-coordinated Pb products at the film surface, consistent with well-established degradation pathways [37]. By contrast, the Cu(BCP)(PEIE)-modified samples retain low Pb 4*f* intensity and negligible binding energy shifts (< 0.1 eV) even after annealing at 100 °C. This persistence of low signal intensity indicates that the interlayer prevents the accumulation of iodine loss-induced under-coordination of Pb and maintains the chemical environment of Pb^2^⁺ against thermal stress. Similar trends were observed in the XPS analysis of the I 3*d* spectra (Fig. S4 and Table S2). In the no-buffer case, Ag reacts with PCBM and mobile halides, producing Ag–I species that appear as clear binding energy downshifts after thermal stress. With a BCP interlayer, the shift is much smaller, indicating partial suppression of iodine migration but still allowing some interfacial reaction. On the contrary, PSCs incorporating Cu(BCP)(PEIE) show a modest upshift (0.5 eV) in I 3*d* binding energy while retaining sharp peak shapes and suppressing extra low-BE features or excess surface Pb/I, consistent with effective passivation rather than degradation. Furthermore, HAADF-STEM EDX mapping (Fig. S5) and XPS analysis in the Cl 2*p* region (Fig. S6) collectively confirm that Cl⁻ anions introduced from the CuCl_2_ precursor remain spatially confined within the buffer layer interface without migrating into the perovskite bulk, rendering their contribution to device degradation negligible. The top-view FE-SEM images (Fig. S7) reveal a non-uniform surface with visible pinholes for the BCP-only buffer layer, as we also observed in AFM images in Fig. S8.

The improved robustness is further examined by water immersion tests (Fig. 2d). Devices incorporating BCP rapidly lose their dark perovskite color within seconds of immersion, signaling accelerated decomposition in humid environments. Conversely, Cu(BCP)(PEIE)-modified devices retain their structural integrity even after 5 min of dipping (Video S1), showing remarkable resistance to water-induced degradation. These results emphasize that the hybrid interlayer provides an effective barrier against both extrinsic (moisture) and intrinsic (ion migration, redox activity) degradation triggers. Moreover, the static water contact angle reveals a progressive increase in hydrophobicity (Fig. 2e). This substantial increase in water repellency is highly beneficial for retarding moisture penetration into the underlying perovskite, thereby contributing to improved environmental stability [38].

Kelvin probe force microscopy (KPFM) measurements were taken to demonstrate the surface potential of the buffer layers under different humidity and temperature conditions. Figure 2f summarizes the average surface potential distribution and corresponding spatial uniformity maps (Fig. S9), with the bar graph indicating that increasing humidity results in a more negative average potential, attributed to water adsorption and dipole formation. For Cu(BCP)(PEIE), the average surface potential represents a 71 mV negative shift from vacuum to high RH, whereas the BCP-based buffer layer exhibits a total negative shift of 97 mV. No-buffer layer (PCBM only) demonstrates minimal variation in surface potential with humidity; however, its higher surface potential results in increased charge extraction barriers compared to Cu(BCP)(PEIE) and BCP-based layers. Cu(BCP)(PEIE) demonstrates superior performance in humid environments by exhibiting a pronounced negative shift in surface potential, which enhances electron injection, as evidenced by uniform surface maps with low spatial variation and absence of defects. Furthermore, Fig. 2g depicts the average surface potential at varying temperatures (− 30, 28, 50, and 70 °C) with the corresponding surface mapping indicated in Fig. S10. All layers exhibit a progressive positive shift with increasing temperature. The streaks observed in the KPFM maps at − 30 °C are instrumental artifacts arising from trace moisture condensation on the cold sample surface and tip–sample contact instability at cryogenic temperatures. Among these, Cu(BCP)(PEIE) displays the least negative potential at low temperature (− 169.67 mV at − 30 °C) and achieves the highest positive value at 70 °C (+ 179.26 mV). This indicates superior thermal responsiveness, the lowest effective work function after moderate thermal annealing, and more efficient surface trap reduction compared to BCP and PCBM. Following the standard KPFM relationship in Eq. 1:

1 $${\Phi}_{\mathrm{s}\mathrm{a}\mathrm{m}\mathrm{p}\mathrm{l}\mathrm{e}} = {\Phi}_{\mathrm{t}\mathrm{i}\mathrm{p}} - e\cdot {V}_{\mathrm{C}\mathrm{P}\mathrm{D}}$$

where e is the elementary charge and ${V}_{CPD}$ is the measured contact potential difference, under a fixed-tip condition, a more positive (less negative) CPD value directly corresponds to a lower sample work function, and vice versa. The progressive positive shift in CPD with temperature corresponds directly to a reduction in the effective surface work function of Cu(BCP)(PEIE), consistent with the macroscopic UPS characterization (Fig. S11), which confirms that the Cu(BCP)(PEIE) interlayer reduces the Ag electrode work function from 4.74 to 4.05 eV, a 0.69 eV decrease that facilitates ohmic contact formation and minimizes the electron extraction barrier at the PCBM/buffer/Ag interface.

Figure 2h illustrates the schematic mechanism to support the aforementioned findings. The complex assembles into a hierarchical molecular network resembling a fishing net function, with Cu^2^⁺ ions serving as weighted nodes, rigid BCP meshes, and flexible PEIE sub-networks integrated throughout (Fig. S12a). HAADF-STEM plane-view imaging and EDX elemental mapping confirm the formation of a continuous, defect-minimized film with homogeneously distributed Cu centers at the nanoscale (Fig. S12b, c), providing structural corroboration for the dense, interconnected interlayer morphology. At the device interface, this fishing-net-inspired interlayer serves multiple functions, simultaneously strengthening interfacial adhesion and blocking moisture ingress, suppressing bidirectional ion migration. When weak interfacial protection is applied, halide ions (*X*-sites) and Ag can migrate across the interface, leading to the formation of AgX, which accelerates perovskite decomposition and interfacial instability [39]. While BCP provides some degree of passivation, it is insufficient to prevent the AgX formation, as previously reported [40]. By contrast, the Cu(BCP)(PEIE) interlayer exerts a dual protective function. The PEIE moieties inhibit ion migration pathways, and Cu–BCP complex stabilizes mobile Ag species, preventing their uncontrolled diffusion. This synergistic effect creates a molecular net that chemically and structurally stabilizes the interfacial environment, minimizes defect formation, blocks detrimental ion exchange, and suppresses electrode-induced degradation to achieve both high stability and improved device performance.

### Photovoltaic Performance and Stability

**Fig. 3:**
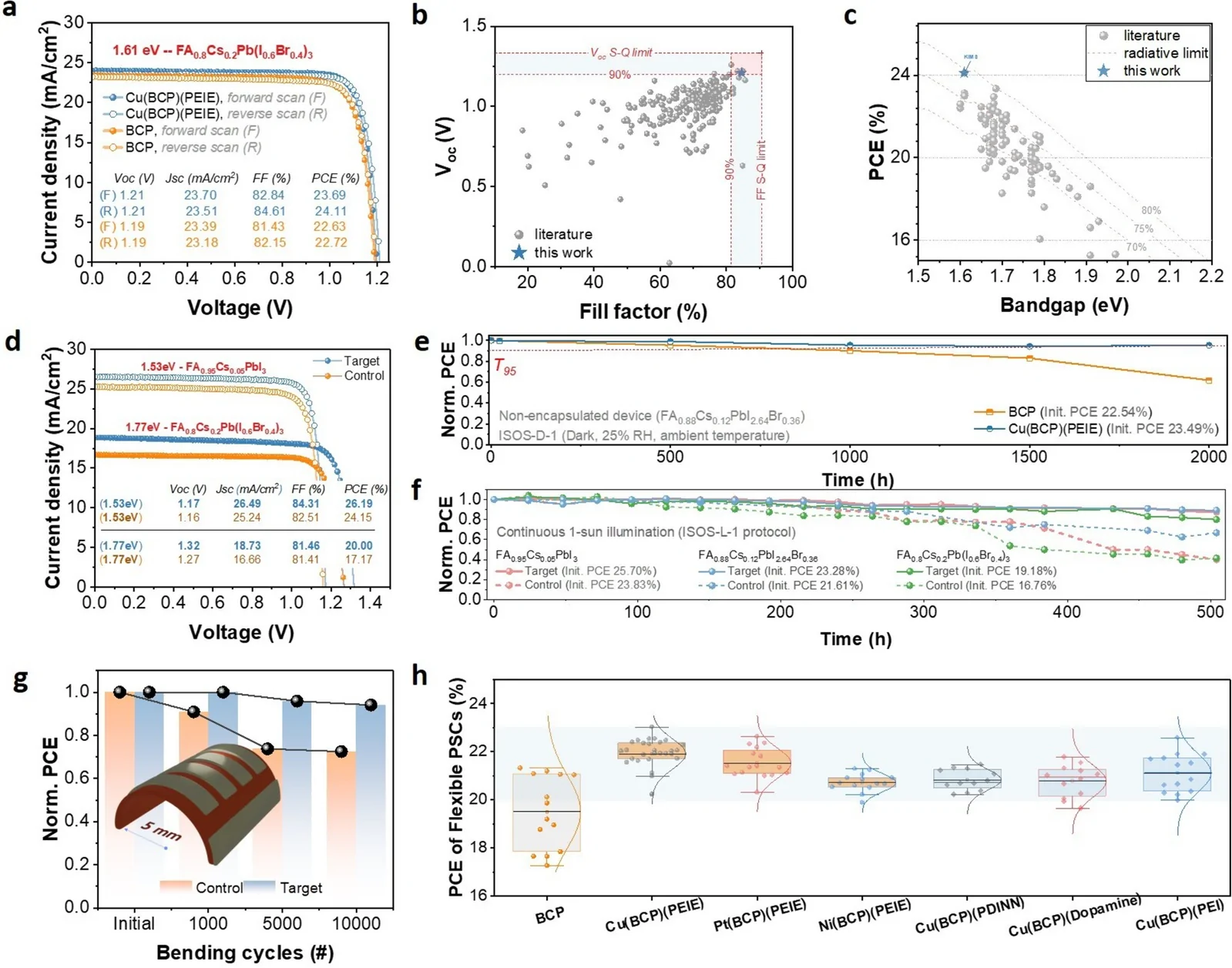
Device performance, stability, and universality of metal-anchored molecular net strategy. **a** *J–V* characteristics of 1.61 eV bandgap PSCs under AM 1.5G illumination. **b** *V*_*oc*_ and *FF* of 1.61 eV PSCs from this work compared to the reported literature and the theoretical S-Q limit. **c** Summary of the reported PCE values with bandgap > 1.6 eV. **d** *J–V* characteristics of 0.048 cm^2^ PSCs for medium (1.53 eV) and wide (1.77 eV) bandgaps. **e** Self-storage stability of PSCs with different interlayers following ISOS-D-1 protocols (25–35% RH, 25 °C). **f** Continuous 1 sun illumination stability of PSCs following ISOS-L-1 protocols. **g** Bending stability of f-PSCs after 10,000 bending cycles. Inset: an illustration of a bending test with a 5 mm bending radius. **h** Statistical PCE distribution of f-PSCs with a set of 15 devices for BCP reference device versus different metal anchors and amine-functionalized polymers

We fabricated an inverted p-i-n PSC to evaluate the functional benefits of the proposed mixed-ligand interfacial complex and firstly tested it on a triple cation mixed halide perovskite film with a bandgap of 1.61 eV (Fig. S13). The current density–voltage (*J–V*) characteristics (Fig. 3a) show that devices incorporating Cu(BCP)(PEIE) achieve a significant enhancement in power conversion efficiency, increasing from 22.72% to 24.11% (certified PCE 24.07%, Fig. S14). The external quantum efficiency (EQE) in Fig. S15 shows a good agreement with that from the *J*–*V* measurement. The resulting fill factor (*FF*) and open-circuit voltage (*Voc*) exceed 90% of the theoretical radiative limit [41], as shown in Fig. 3b, placing these devices among the highest performing reported PSCs for a > 1.60 eV bandgap (Fig. 3c and Table S3). This performance enhancement arises from the synergistic interplay between the Cu–BCP coordination, which reduces the electron injection barrier, and the PEIE component, which establishes favorable interfacial dipoles that enhance band alignment with the perovskite absorber. To systematically evaluate the contributions of each component, we fabricated PSCs with varying buffer layer configurations, as detailed in Table S4. The introduction of PEIE into the Cu-BCP complex resulted in a notable increase in *FF*. In contrast, the use of a CuCl_2_ interlayer led to a significant reduction in both *Voc* and *FF*, primarily due to shunting pathways between the Ag electrode and PCBM, coupled with energy-level misalignment. Additionally, by varying the CuCl_2_-to-BCP ratio (Table S5), we determined that the optimal CuCl_2_-to-BCP ratio was 1:10 (v/v), as higher Cu content led to declines in *Voc* and *FF* (Fig. S16). Furthermore, the thickness-dependent performance of the buffer layer was investigated by varying the PEIE concentration in the Cu-BCP complex (Table S6) and buffer layer spin speed (Table S7). Optimal device performance was achieved at a buffer layer thickness of ~ 9 nm using 0.025 wt% PEIE at 4000 rpm, which minimized shunting pathways and improved film uniformity.

We assessed the versatility of the Cu(BCP)(PEIE) interlayer across different perovskite bandgap and diverse processing conditions and substrates. Figure 3d presents representative *J–V* characteristics of reference and Cu(BCP)(PEIE)-treated cells using 1.53 eV perovskite FA_0.95_Cs_0.05_PbI_3_ and 1.77 eV perovskite FA_0.8_Cs_0.2_Pb(I_0.6_Br_0.4_)_3_ with the statistics of device performance metrics for each bandgap is depicted in Fig. S17. The resulting photovoltaic performance of Cu(BCP)(PEIE)-based device reveals a PCE of 26.19% (certified 25.18%, Fig. S18) and 20.00% (certified 19.19%, Fig. S19), for 1.53 eV and 1.77 eV bandgap, respectively. Moreover, the *Voc* and *FF* for both different bandgaps are approaching 90% of the thermodynamic limit (Fig. S20). We also observed that, across all bandgaps, the approach not only increased *Voc* and *FF* but also slightly enhanced *Jsc*. To verify this *Jsc* improvement, external quantum efficiency (EQE) measurements were taken on devices with 1.53 eV and 1.77 eV bandgaps (Fig. S21). The integrated *Jsc* values showed excellent agreement with those extracted from the *J*–*V* curves, confirming the universality of the strategy in reducing the electron injection barrier. Moreover, we fabricated 1 cm^2^ rigid 1.61 eV PSCs (Table S8), in which the metal-anchored coordination network substantially mitigated efficiency losses during scaling, reducing the PCE drop from small- to large-area devices to 7.8% for Cu(BCP)(PEIE), compared with 15.7% for BCP. This improvement primarily arises from the preservation of high FF and *V*_oc_. Furthermore, 1 cm^2^ air-processed devices (30–50% RH, 18–25 °C) fabricated on both rigid (Fig. S22 and Table S9) and flexible substrates (Fig. S23) consistently outperformed their BCP-only counterparts, demonstrating the interlayer’s scalability and compatibility with flexible and ambient-processed PSCs. Notably, the air-processed flexible devices achieved a PCE of 23.03%, with an *FF* of up to 81.75%, again placing them among the best reported to date (Table S10).

To gain deeper insight into the protective function of the mixed-ligand complex toward the PSCs, we conducted ISOS-D-1 shelf-life measurements on unencapsulated devices stored under ambient conditions (25–35% RH, 25 ± 2 °C) as shown in Fig. 3e. Devices incorporating the proposed buffer layer degraded much more slowly than those with the BCP interlayer, retaining 95% of their initial PCE even after over 2000 h of storage. Operational stability was further assessed under continuous 1 sun illumination (ISOS-L-1) for unencapsulated devices with different perovskite bandgaps. As shown in Fig. 3f, BCP-based devices showed a gradual efficiency decay across all bandgap compositions, whereas devices incorporating Cu(BCP)(PEIE) sustained more than 90% of their initial efficiency beyond 500 h. The maximum power point tracking (MPPT) confirming that Cu(BCP)(PEIE) devices exhibit stable power output at the MPP with minimal degradation or burn-in losses over the tracking period (Fig. S24). We conducted XPS analysis of the Cu 2*p* core level before and after 200 h of aging test (continuous 1 sun illumination at 40 °C) to probe the chemical integrity of the buffer layer under operational stress (Fig. S25). Both pristine and aged perovskite-based Cu(BCP)(PEIE) film exhibits a well-defined Cu 2*p*_3/2_ peak at ~ 930 eV, confirming that Cu^2^⁺ remains in its divalent, coordinated state throughout the operational stability test. The markedly extended lifetimes under both shelf storage and continuous 1 sun illumination indicate that the mixed-ligand complex effectively suppresses ion migration and alleviates interface-driven degradation.

The devices were additionally subjected to a damp heat stress test (85 °C, 85% RH; Fig. S26), during which they retained 80% of their initial PCE after 600 h. Performance restoration after thermal treatment was further assessed (Fig. S27) by exposing perovskite solar cells to short-term thermal treatment at various temperatures (85, 100, 120, and 150 °C for 10 min). Heating to temperatures ≤ 100 °C resulted in a modest increase in PCE, which we attribute to thermally induced lattice relaxation and the healing of trap states commonly observed in perovskite materials [42]. At higher temperatures (120 and 150 °C), however, devices incorporating BCP exhibited substantial efficiency losses, whereas Cu(BCP)(PEIE)-based devices retained approximately 80% of their initial performance. To better understand the origin of this thermally induced PCE gain, we deliberately immersed the complete devices in deionized water for 5 min, followed by annealing at 100 °C (Fig. S28 and Table S11 and S12). Remarkably, the Cu(BCP)(PEIE) revealed a PCE enhancement when multiple post-immersion thermal treatments were performed, whereas reference cells containing only BCP showed no comparable performance restoration after thermal treatment. We reasoned this behavior as evidence of moisture-induced partial *α*-to-*δ* phase transition with thermally assisted partial recovery, coupled with the superior moisture-blocking ability and interfacial defect passivation provided by the Cu(BCP)(PEIE) layer (Fig. S29). These features appear to suppress irreversible decomposition pathways and enable efficient charge extraction even after water exposure. We also studied the water immersion of FA_0.95_Cs_0.05_PbI_3_ and FA_0.8_Cs_0.2_Pb(I_0.6_Br_0.4_)_3_ perovskite as indicated in Fig. S30. The PSCs with different bandgaps exhibit a distinguishable retained PCE after a few times of 5 min water soaking.

Figure 3g examines the mechanical durability through cyclic bending tests (bending radius of 5 mm). The measurements were taken in ambient air (relative humidity of 30–50%, temperature of 20–25 °C). The Cu(BCP)(PEIE) devices maintain 95% of their initial PCE after 10,000 bending cycles, whereas the control (BCP) devices retain only ~ 70%. The air storage stability of *f*‑PSCs was also investigated, and as shown in Fig. S31, non‑encapsulated devices based on Cu(BCP)(PEIE) maintained over ~ 80% of their initial PCE after ~ 500 h in air (30–60% RH, ~ 25 °C). In contrast, BCP‑based devices degraded to below ~ 60% under the same conditions, highlighting the superior interfacial robustness and ambient stability imparted by the Cu(BCP)(PEIE) layer. Furthermore, by varying both the metal centers and amine-functionalized polymers in f-PSCs (Fig. S32), we achieve higher performance than the BCP-only reference device (Fig. 3h). The selection principle for the metal node in the ternary framework is based on three factors: (1) coordination affinity toward amine and phenanthroline backbone, (II) coordination geometry and d-orbital occupancy, and (III) electrochemical stability under the device operating potential window. Cu^2^⁺ demonstrates the optimal balance of coordination stability, work function modification, and solution processability. In comparison, Pt^2^⁺ exhibits different coordination kinetics and stronger metal–ligand bonding, which may suppress the dynamic molecular rearrangement needed for effective polymer sub-network formation. Ni^2^⁺ shows weaker coordination affinity toward the BCP phenanthroline due to its lower crystal field stabilization energy in this ligand environment. Moreover, the water immersion test of cells with different compositions reveals markedly enhanced operational stability, highlighting the broad applicability (Fig. S33).

### PSCs under Low-Light Intensity and Extreme Conditions

**Fig. 4:**
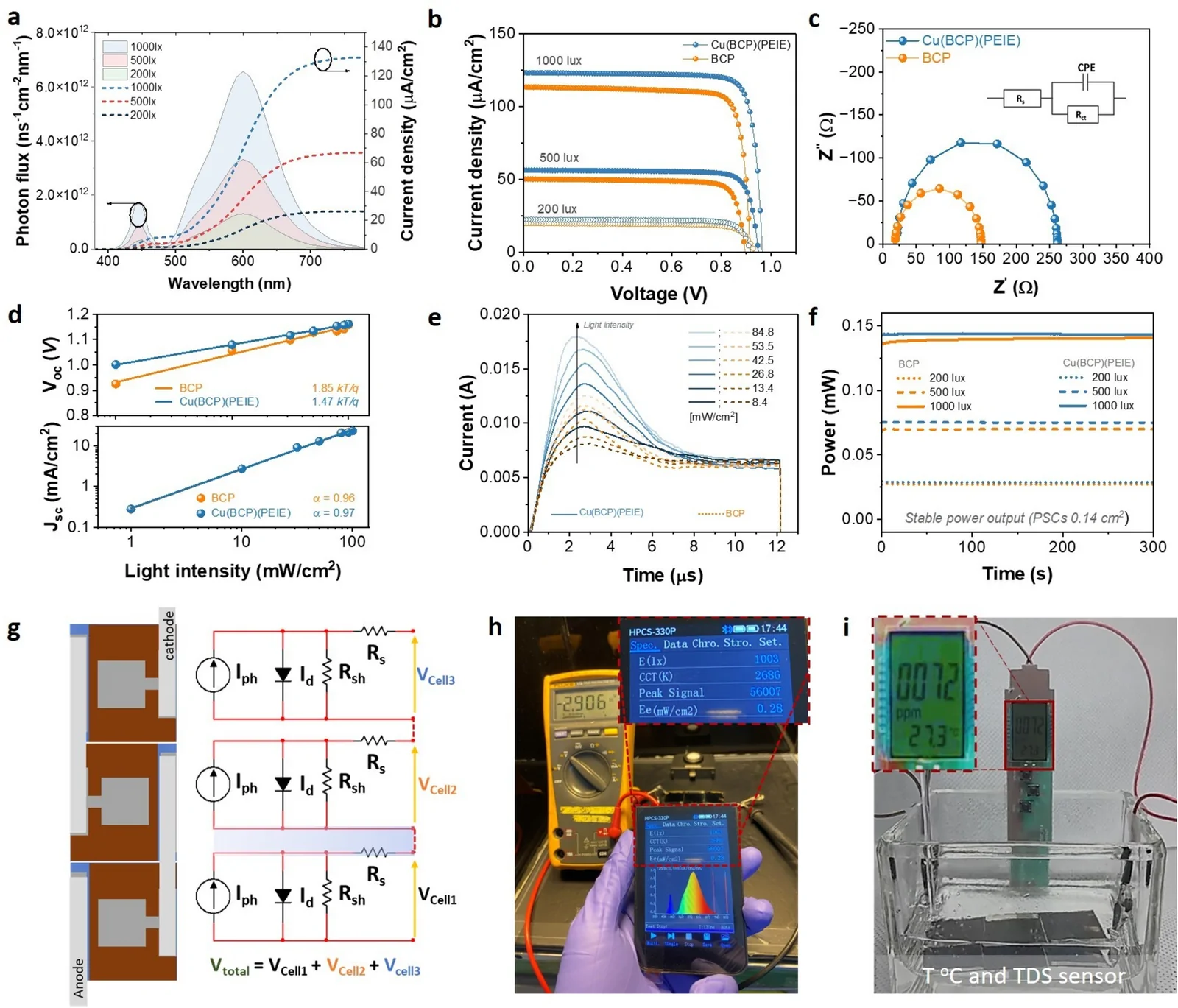
Energy harvesting in varied low-light for extreme conditions applications. **a** Photon flux and integrated current density spectra of warm LED light (2700 K) at 200, 500, and 1000 lx illumination intensities. **b** *J–V* curves characteristic of BCP- and Cu(BCP)(PEIE)-based interlayer at 1000, 500, and 200 lx, respectively. **c** Nyquist plot with a bias voltage of 1 V in dark conditions. **d** *V*_oc_ (top) and *J*_sc_ (bottom) dependency as a function of light intensities. **e** Photo-CELIV curves of PSCs measured under various light illuminations. **f** Stabilized power output of perovskite solar cells (0.14 cm^2^) as a function of illuminance. **g** Schematic of an electrical circuit from the three 1 cm^2^ PSCs connected in series. **h** Accumulated voltage from three cells connected in series under low indoor light (~ 1000 lx). **i** Utilization of perovskite solar cells as a power source under low indoor light while immersing the unencapsulated devices in deionized water for TDS and temperature sensor

As the low charge recombination and suppressed leakage current are among the crucial points for high-performance low-light energy harvesting, this study further utilizes the perovskite solar cells in indoor photovoltaic (IPV) under harsher conditions. The custom-designed IPV measurement setup is illustrated in Fig. S34, in which calibrated dual-sensor monitoring ensured accurate light intensity calibration and minimized spectral/angular mismatches. Theoretical current densities were calculated by integrating the photon flux spectra with their corresponding illumination intensities, as shown in Fig. 4a.

Figure 4b shows the *J–V* characteristics of rigid devices (active area 0.048 cm^2^) under varying illumination intensities of 200, 500, and 1000 lx. The performance advantage of the engineered interface persisted, underscoring its robustness across a wide dynamic range of indoor conditions (Table S13). Notably, the proposed approach can maintain high *FF* under low-intensity illumination and exhibits one of the highest *FF*s for indoor light perovskite solar cells (Table S14), especially under 500 lx illumination. Nyquist plots (Fig. 4c) reveal significantly larger recombination resistance (*R*_rec_) for Cu(BCP)(PEIE) compared to pristine BCP. The semicircle broadening and higher fitted *R*_rec_ values suggest suppressed non-radiative recombination at the ETL/metal interface, attributable to effective passivation of interfacial traps by Cu–N coordination and PEIE dipoles. This strongly suggests that the mixed-ligand complex effectively mitigates trap-mediated recombination at the interface, in line with the light intensity dependence (Fig. 4d) and charge recombination analysis (Fig. S35). Photo-CELIV measurements (Fig. 4e) further reveal that Cu(BCP)(PEIE)-based devices exhibit faster charge extraction and stronger photocurrent transients compared to BCP reference interlayers (Table S15). Stabilized power output under varying illuminance (Fig. 4f) confirms that Cu(BCP)(PEIE) devices deliver higher absolute power across all tested intensities, maintaining stable maximum power point operation—a critical attribute for indoor energy harvesting under low and fluctuating light.

Voltage accumulation studies of three 1 cm^2^ PSCs connected in series (Fig. 4g) demonstrate scalable output and a simple circuit design suitable for powering low-power electronics under weak indoor lighting (Figs. 4h and S36). In a proof-of-concept demonstration, these series-connected, unencapsulated PSCs successfully powered a total dissolved solids (TDS) and temperature sensor under indoor lighting (Fig. 4i; Video S2) even when submerged in water, and remained functional for several min. Fig. S37 shows stable operation across immersion depths of 1–5 cm, with minor performance improvements attributed to reduced reflection losses in water [43]. To assess the environmental safety of direct water immersion, inductively coupled plasma mass spectrometry (ICP–MS) analysis was performed on the water after the immersion test to quantify leaked Pb and Cs species (Fig. S38). The control device without a buffer layer showed the highest Pb^2+^ concentration in water (approximately 0.77 ppm), while the BCP-based and Cu(BCP)(PEIE)-based devices exhibited reduced Pb^2+^ concentrations of about 0.63 and 0.49 ppm, respectively. For Cs^+^, the no-buffer and BCP devices showed concentrations of roughly 10–11 ppm, whereas the Cu(BCP)(PEIE)-based device exhibited Cs^+^ leakage near the background level, indicating much stronger suppression of perovskite component dissolution. These results demonstrate that the Cu(BCP)(PEIE) interlayer significantly enhances resistance to water-induced degradation and ion leakage. Nevertheless, because detectable Pb leakage remains after immersion, the present results should be interpreted as evidence of improved water tolerance rather than complete environmental safety for direct underwater application.

## Conclusions

In this work, we report a multifunctional metal-anchored molecular net cathode interlayer, Cu(BCP)(PEIE), that integrates a netlike physical architecture with Cu–N coordination chemistry and amine-induced dipole engineering to simultaneously enhance efficiency and stability in perovskite solar cells. Coordination among Cu^2^⁺ ions, BCP, and PEIE improves chemical robustness, suppresses interfacial ion migration, and increases hydrophobicity, while the dipolar effect of PEIE lowers the cathode work function, facilitating efficient electron extraction and reducing interfacial recombination. Devices incorporating the Cu(BCP)(PEIE) interlayer exhibit high efficiency, excellent reproducibility, and long-term operational stability, retaining over 95% of their initial performance after 2000 h of air storage and demonstrating extended lifetimes under continuous operation. Notably, the devices show partial phase recovery after water immersion followed by thermal treatment, indicating intrinsic interfacial resilience. The metal anchor and polymer components can be modularly substituted with other transition metals and amine-functionalized polymers, underscoring the generality of this design. Together, these results establish the molecular net strategy as a broadly applicable interfacial paradigm for achieving chemically durable and high-performance optoelectronic devices.

## Supplementary Information

Below is the link to the electronic supplementary material.

Supplementary file1 (MP4 1457 KB)

Supplementary file2 (MP4 2890 KB)

Supplementary file3 (DOCX 12323 KB)
