# Supplementary material for: Air-Processed and Water-Stable Perovskite Solar Cells Enabled by a Fishing-Net-Inspired Interfacial Network
Source: Nanomicro Lett. 2026 Jul 17;18:440. doi: 10.1007/s40820-026-02295-5 (PMC13376114; doi:10.1007/s40820-026-02295-5)
Supplement: Supplementary file 3 — Supplementary file3 (DOCX 12323 KB) [file 40820_2026_2295_MOESM3_ESM.docx]

Supplementary Information for

**Air-Processed and Water-Stable Perovskite Solar Cells Enabled by a Fishing-Net-Inspired Interfacial Network**

Muh Fadhil Albab^1,2^, Muhammad Jahandar^1^, Ah Ra Kim^1^, Jinhee Heo^3^, Yong Hyun Kim^2^, Youngkyoo Kim^4,5^, Gi-Hwan Kim^6^, Ji-Youn Seo^7^, Shinuk Cho^8^, Soyeon Kim^1*^, and Dong Chan Lim^1,5*^

^1^Energy and Environment Materials Research Division, Korea Institute of Materials Science (KIMS); Changwon 51508, Republic of Korea

^2^Department of Smart Green Technology Engineering, Pukyong National University; Busan 48513, Republic of Korea

^3^Materials Testing & Reliability Division, Korea Institute of Materials Science (KIMS); Changwon 51508, Republic of Korea

^4^Organic Nanoelectronics Laboratory and KNU Institute for Nanophotonics Applications (KINPA) Department of Chemical Engineering Kyungpook National University; Daegu 41566, Republic of Korea

^5^Semiconductor Unit, Institute for Advanced Technology Convergence, Kyungpook National University; Daegu 41566, Republic of Korea

^6^School of Materials Science and Engineering, Gyeongsang National University; Jinju 52828, Republic of Korea

^7^Department of Nano Fusion Technology, Pusan National University; Busan 46241, Republic of Korea

^8^Department of Semiconductor Physics & Engineering and Energy Harvest Storage Research Center, University of Ulsan; Ulsan, 44610, Republic of Korea

*Corresponding authors. E-mail: [dclim@kims.re.kr](mailto:dclim@kims.re.kr) (Dong Chan Lim); [kimso1965@kims.re.kr](mailto:kimso1965@kims.re.kr) (Soyeon Kim)

**Supplementary Figures and Tables**


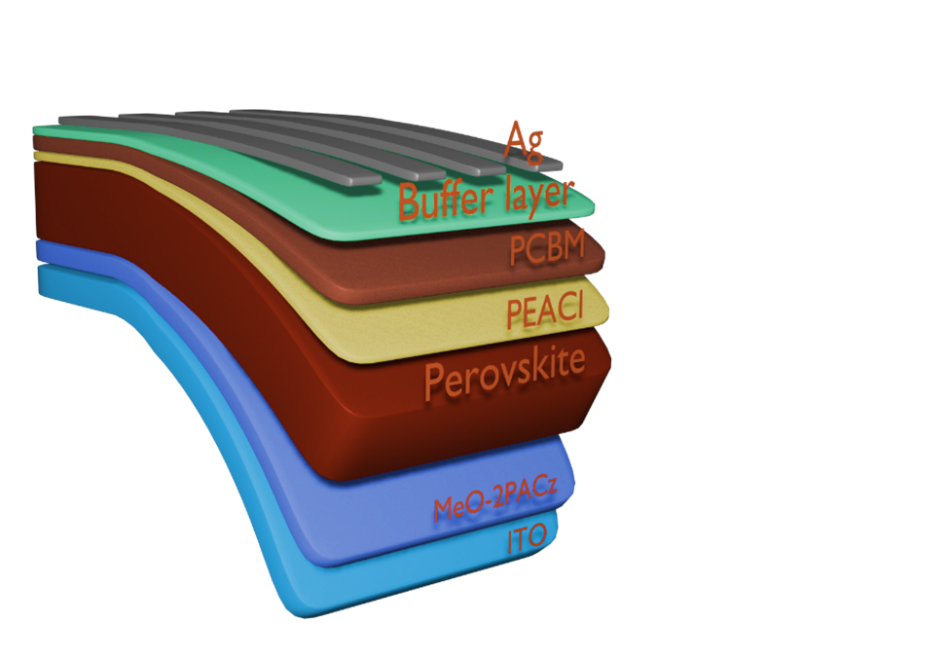


**Fig. S1** A schematic of the perovskite structure (p-i-n), layers, and configuration


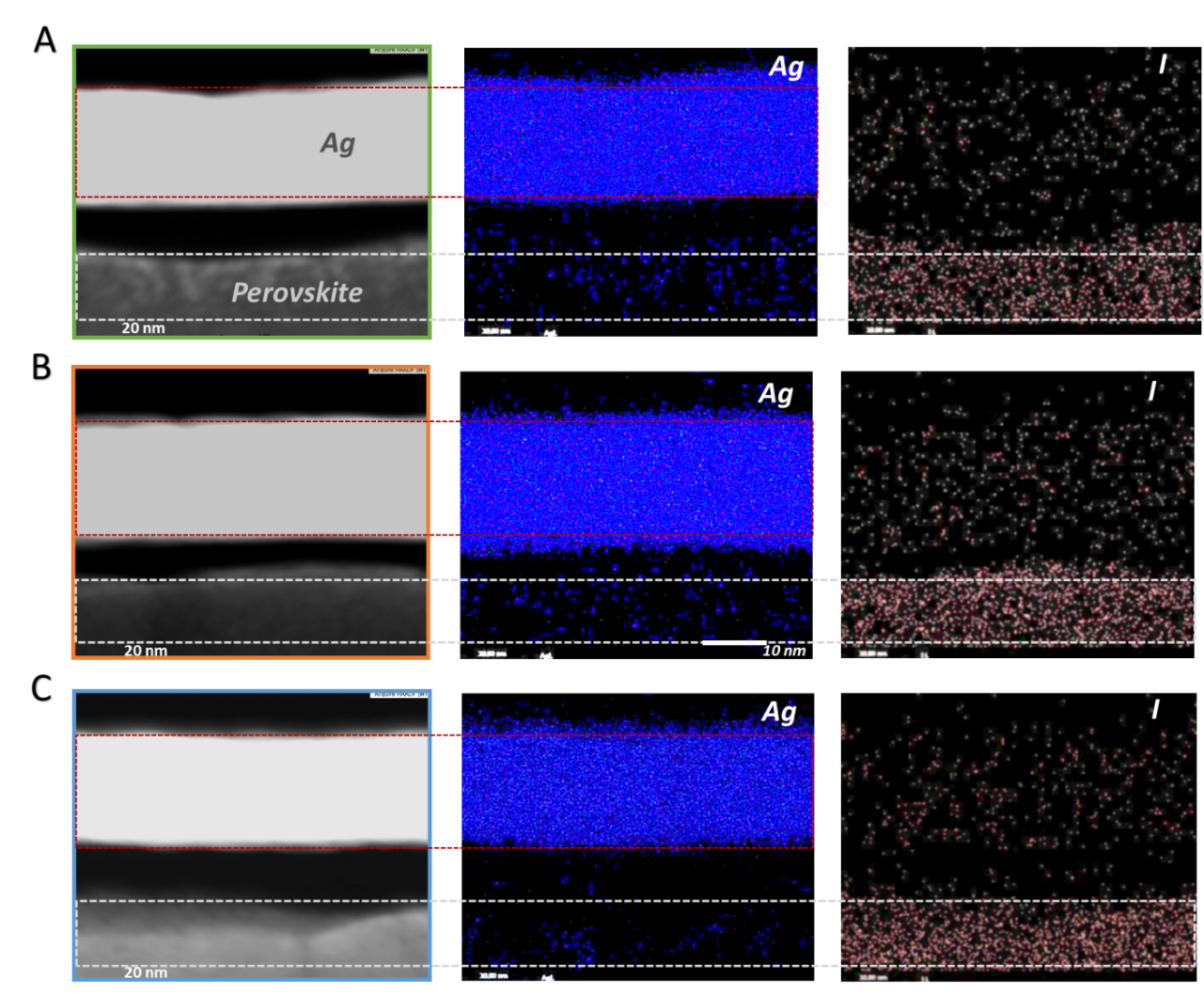


**Fig. S2** High-angle annular dark-field scanning transmission electron microscopy (HAADF-STEM) images and the energy dispersive X-ray spectroscopy (EDX) maps of Ag and I elements after ageing at 100 °C for 48h in ambient air. (a) perovskite solar cells without a buffer layer and with (b) BCP and (c) Cu(BCP)(PEIE) interlayer.


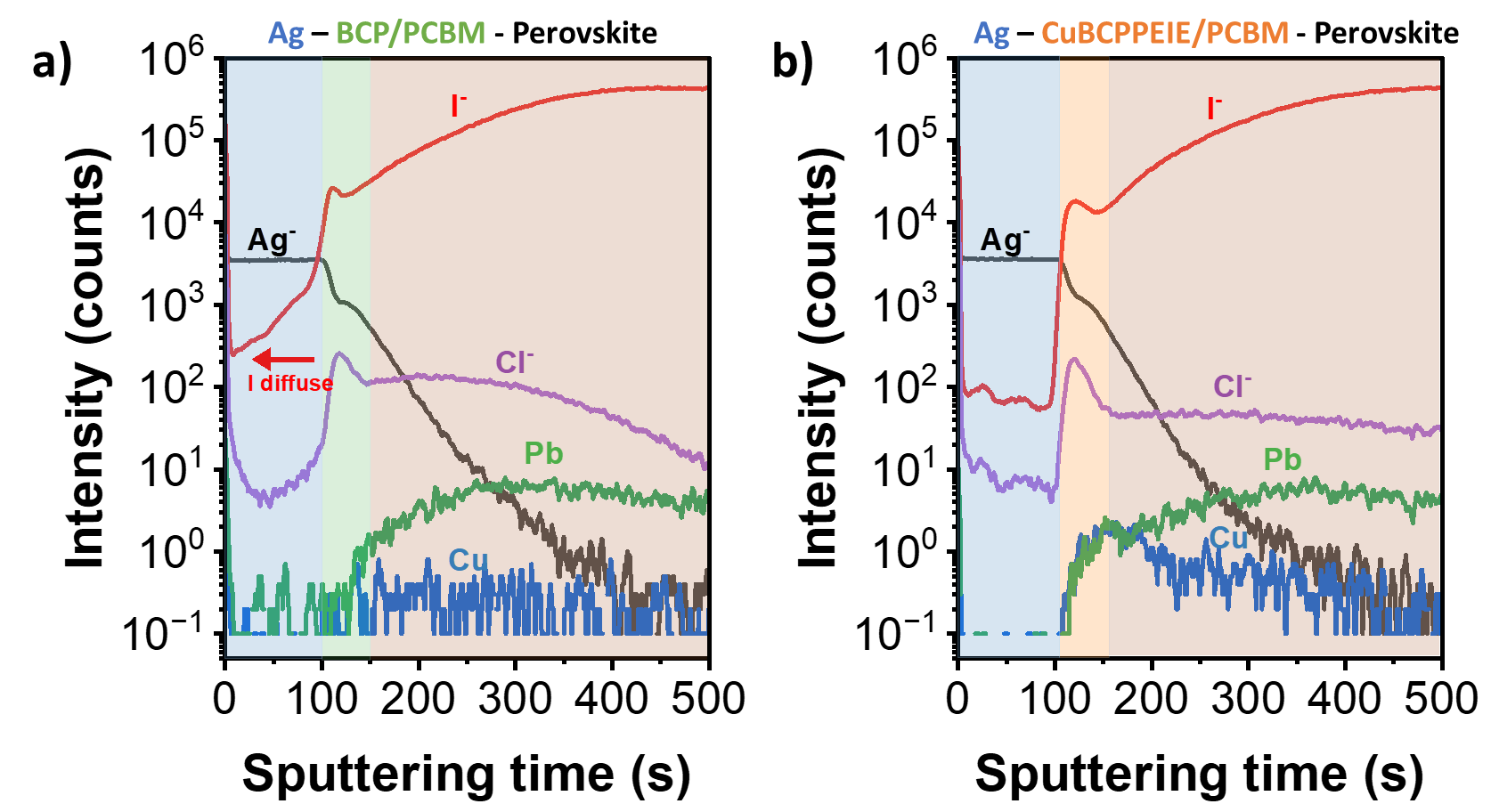


**Fig. S3** The in-depth profile of ToF-SIMS of (a) BCP-based and (b) Cu(BCP)(PEIE)-based device after thermally aged at 100 ^o^C for 48h.


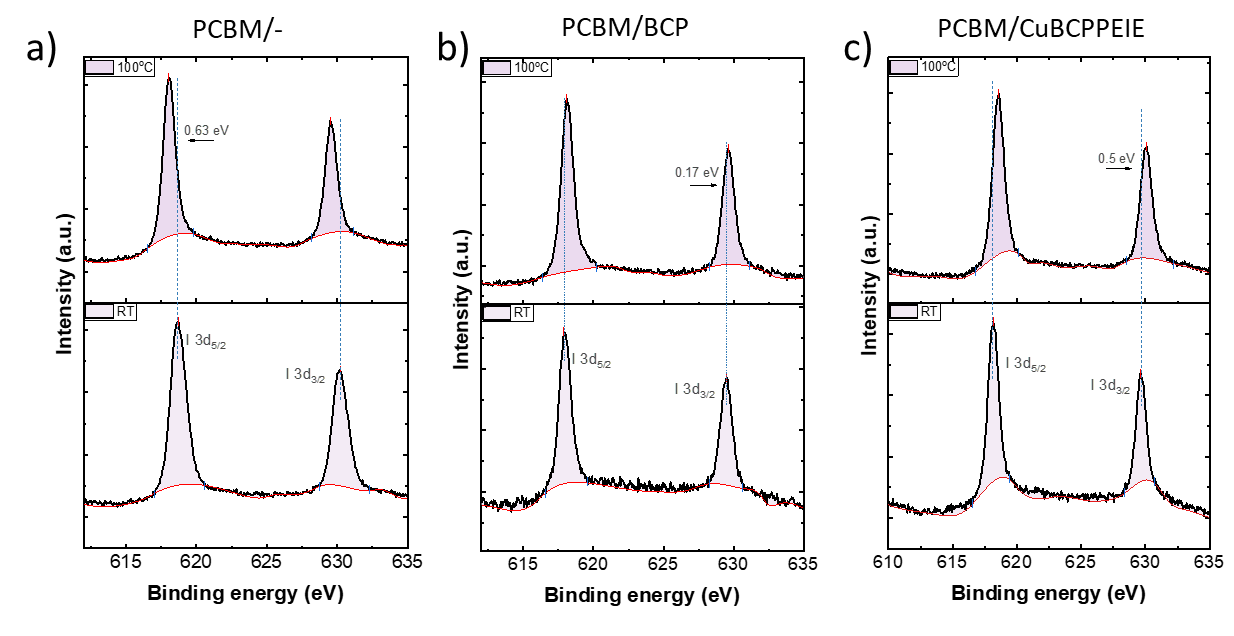


**Fig. S4** XPS spectra of FA_0.88_Cs_0.12_PbI_2.64_Br_0.36_/PCBM/with or without buffer layer/5nm Ag, after degradation test for 48 hours at RT and 100 ^o^C. a-c the I 3d XPS peak of PCBM, PCBM with BCP, and PCBM with Cu(BCP)(PEIE), respectively.


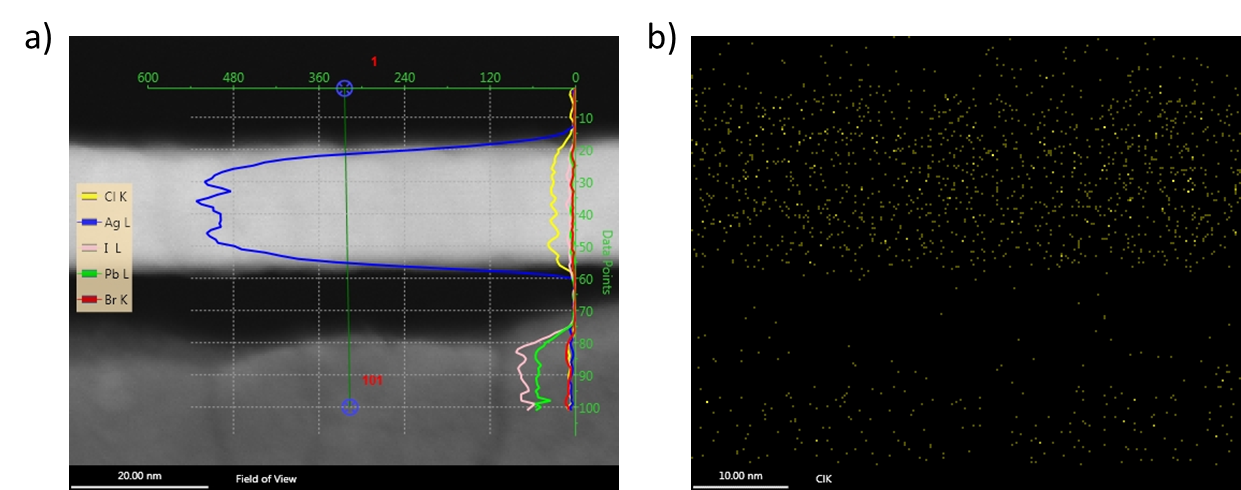


**Fig. S5** a) HAADF-STEM and EDX elemental maps of perovskite solar cells and corresponding line profile. b) EDX elemental maps of Cl element after ageing at 100 °C for 48h in ambient air.

**
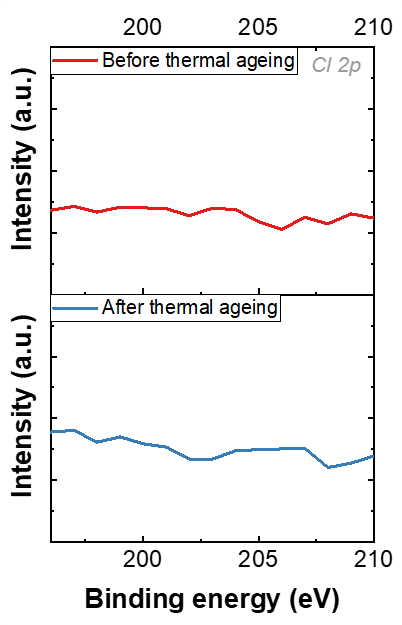
**

**Fig. S6** The XPS of Cl 2p before and after thermal ageing (100 ^o^C, 48h in ambient air).


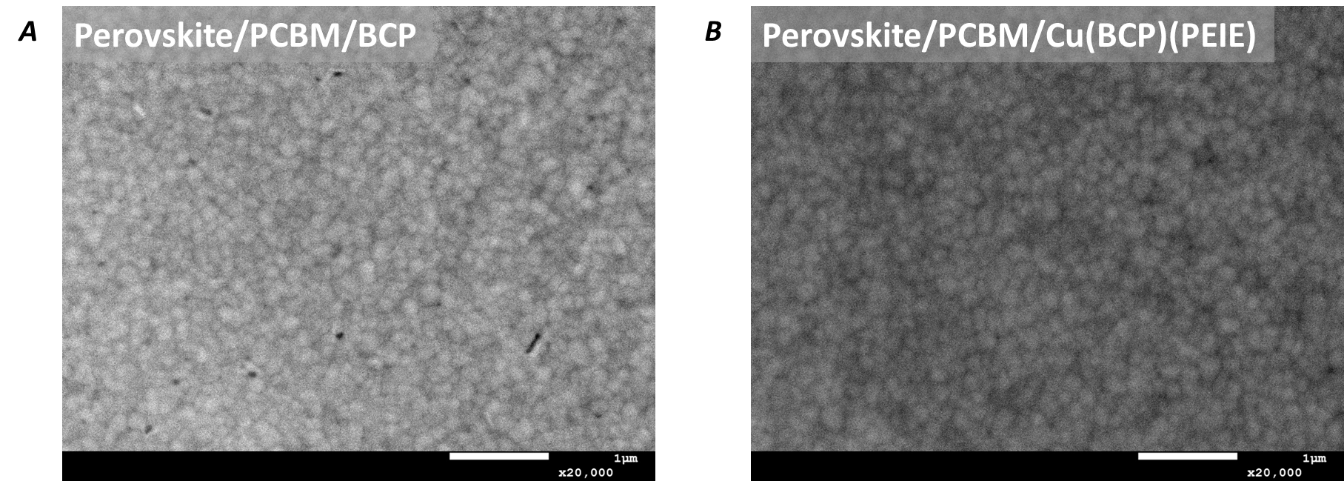


**Fig. S7** SEM images of Perovskite/PCBM. a) BCP and b) Cu(BCP)(PEIE) buffer layer


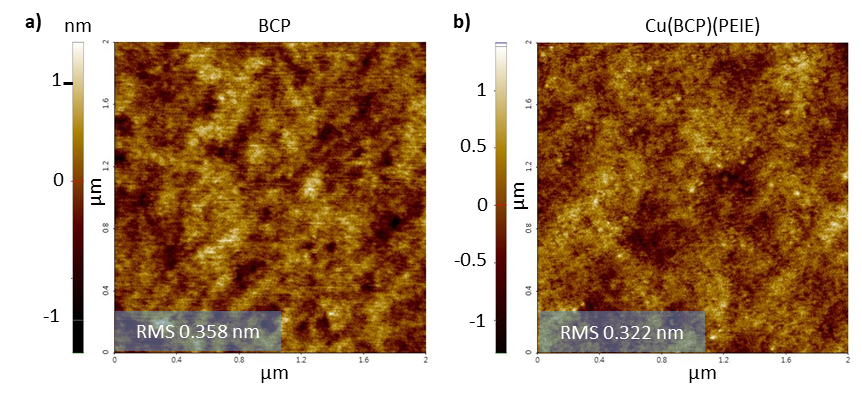


**Fig. S8** AFM images of PCBM with a) BCP and b) Cu(BCP)(PEIE) buffer layer


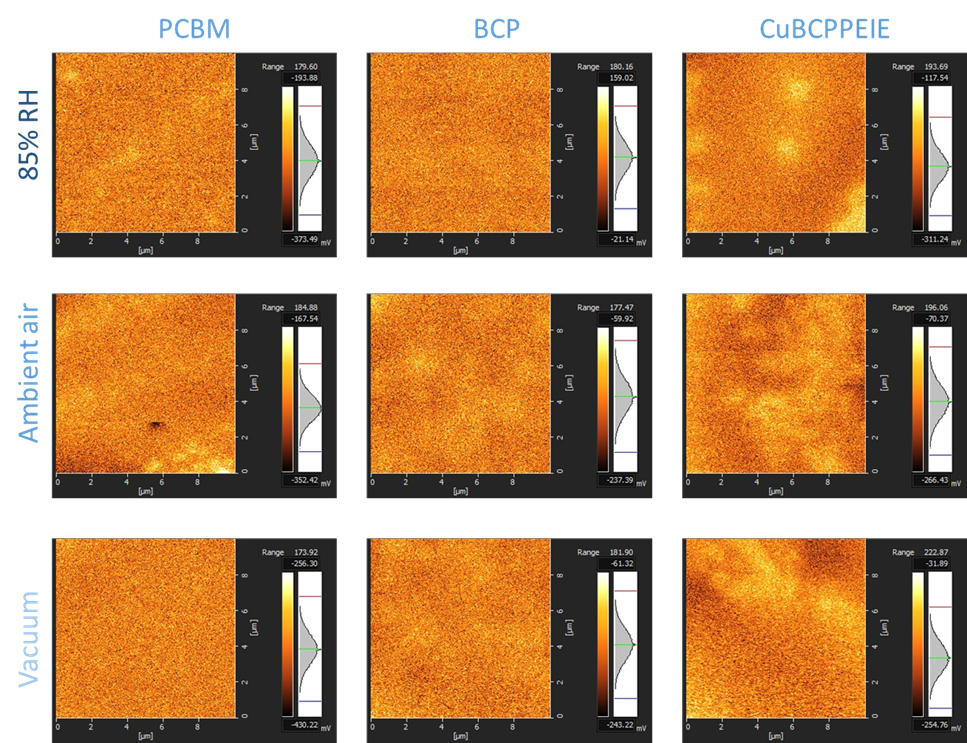


**Fig. S9** Surface potential of different buffer layers with humidity level


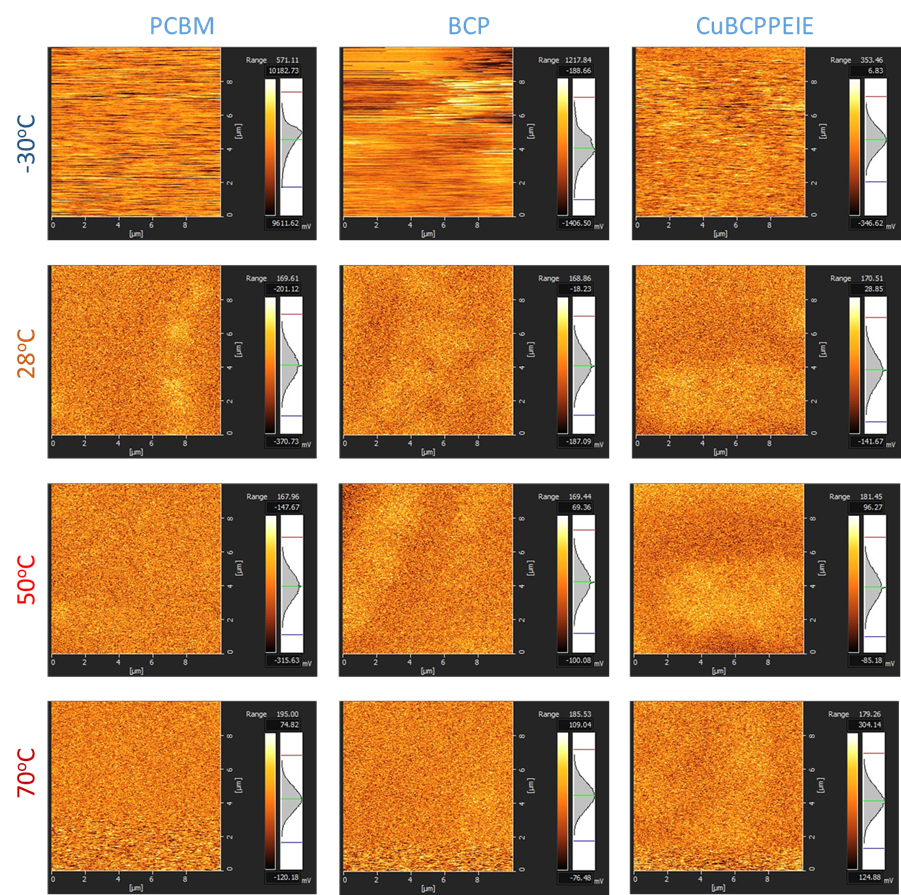


**Fig. S10** Surface potential of different buffer layers with temperature


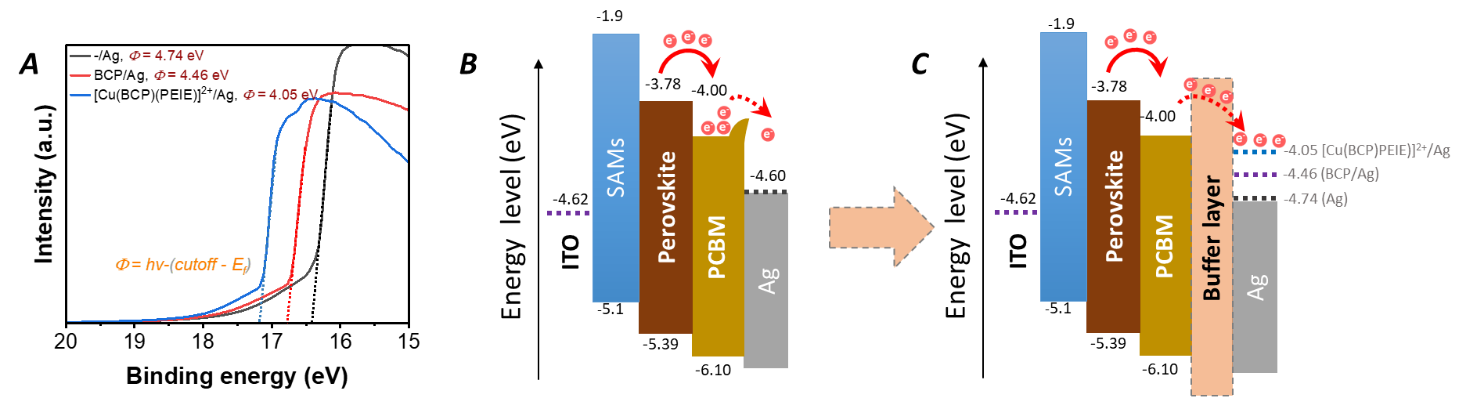


**Fig. S11** UPS measurement results of different buffer layer. a) The UPS plot of Ag without and with different cathode buffer layers indicates a progressive reduction in the silver (Ag) work function of 0.7 eV, which aligns with the formation of a strong interfacial dipole. Such a dipole is attributed to amine and ethoxy groups in polyethylenimine ethoxylated (PEIE) and copper-nitrogen (Cu–N) coordination, both of which orient positive charge toward the metal. A schematic illustration of Ag energy level alignment without b) cathode buffer layer and c) with BCP and Cu(BCP)(PEIE) buffer layer.


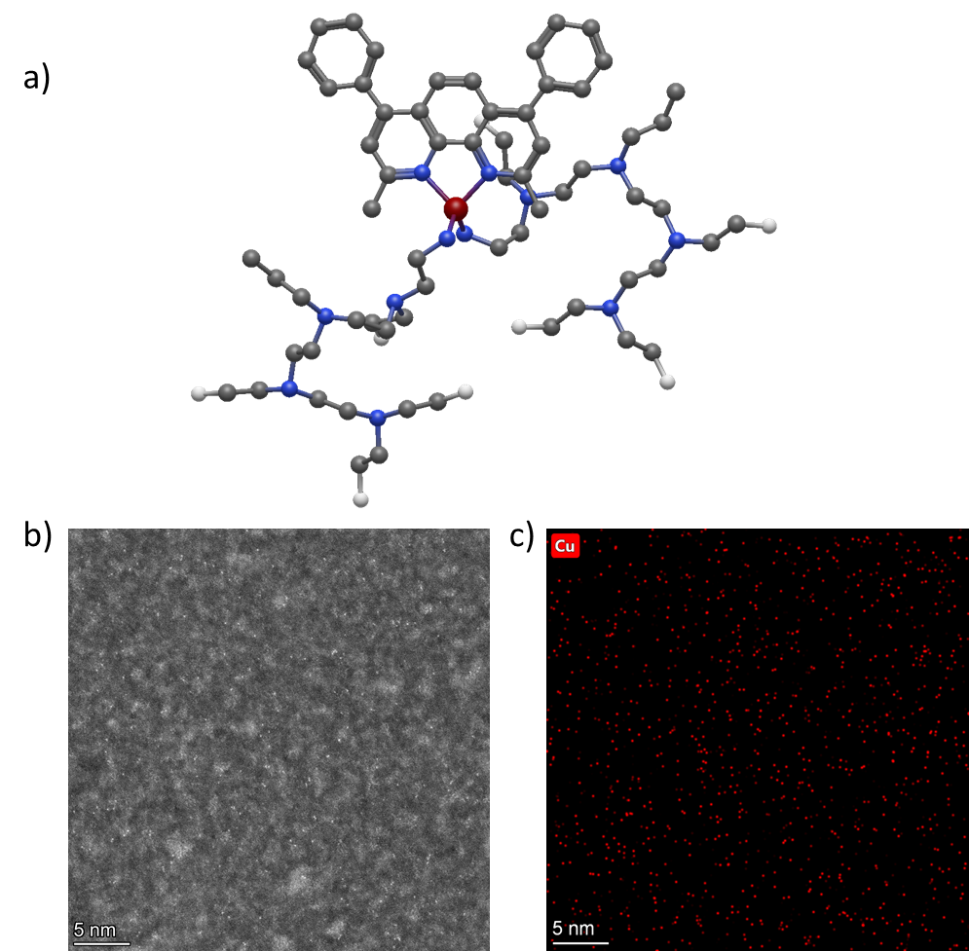


**Fig. S12** a) Molecular geometry optimization of the Cu(BCP)(PEIE) ternary complex (85 atoms; Avogadro energy minimization) reveals a Cu–N coordination node bridging the rigid BCP scaffold and flexible PEIE sub-chains. b) High-angle annular dark-field scanning transmission electron microscopy (HAADF-STEM) image of Cu(BCP)(PEIE) plane view. c) EDX elemental analysis of Cu(BCP)(PEIE) indicating the present of metal-anchored (Cu) on the surface.

**
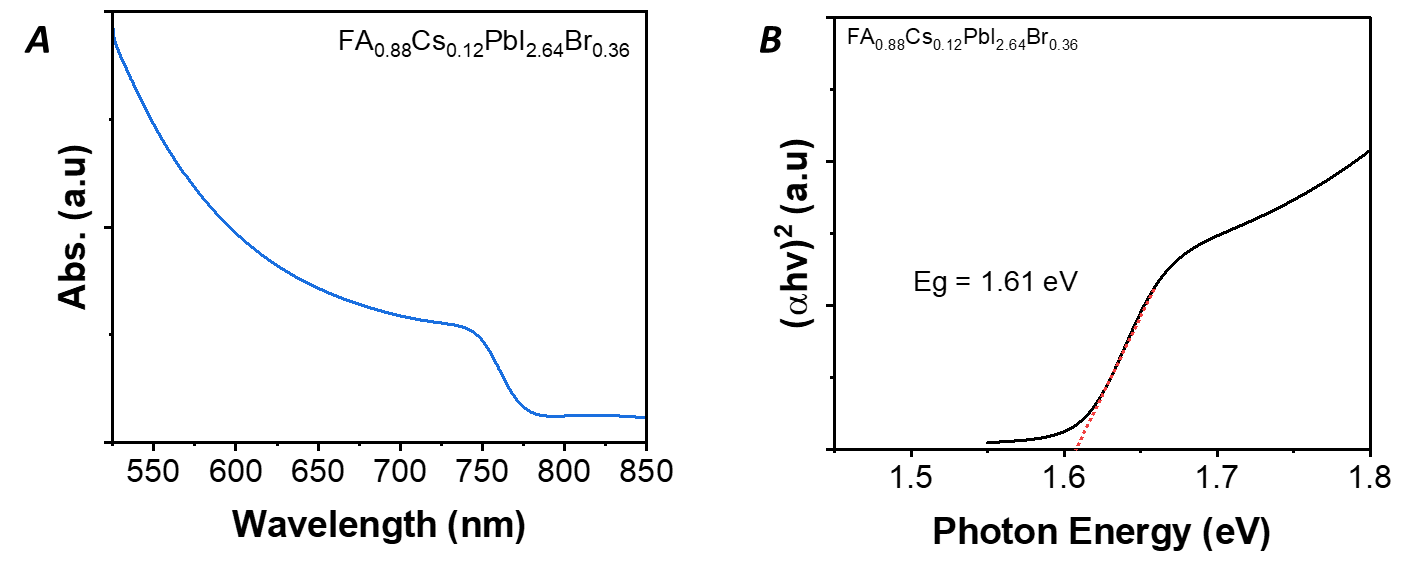
**

**Fig. S13** Absorbance and bandgap calculation. (A) Absorbance spectra with the absorption edge and (B) band-gap derived from tauc plot of triple cation mixed halides perovskite used mainly in this study


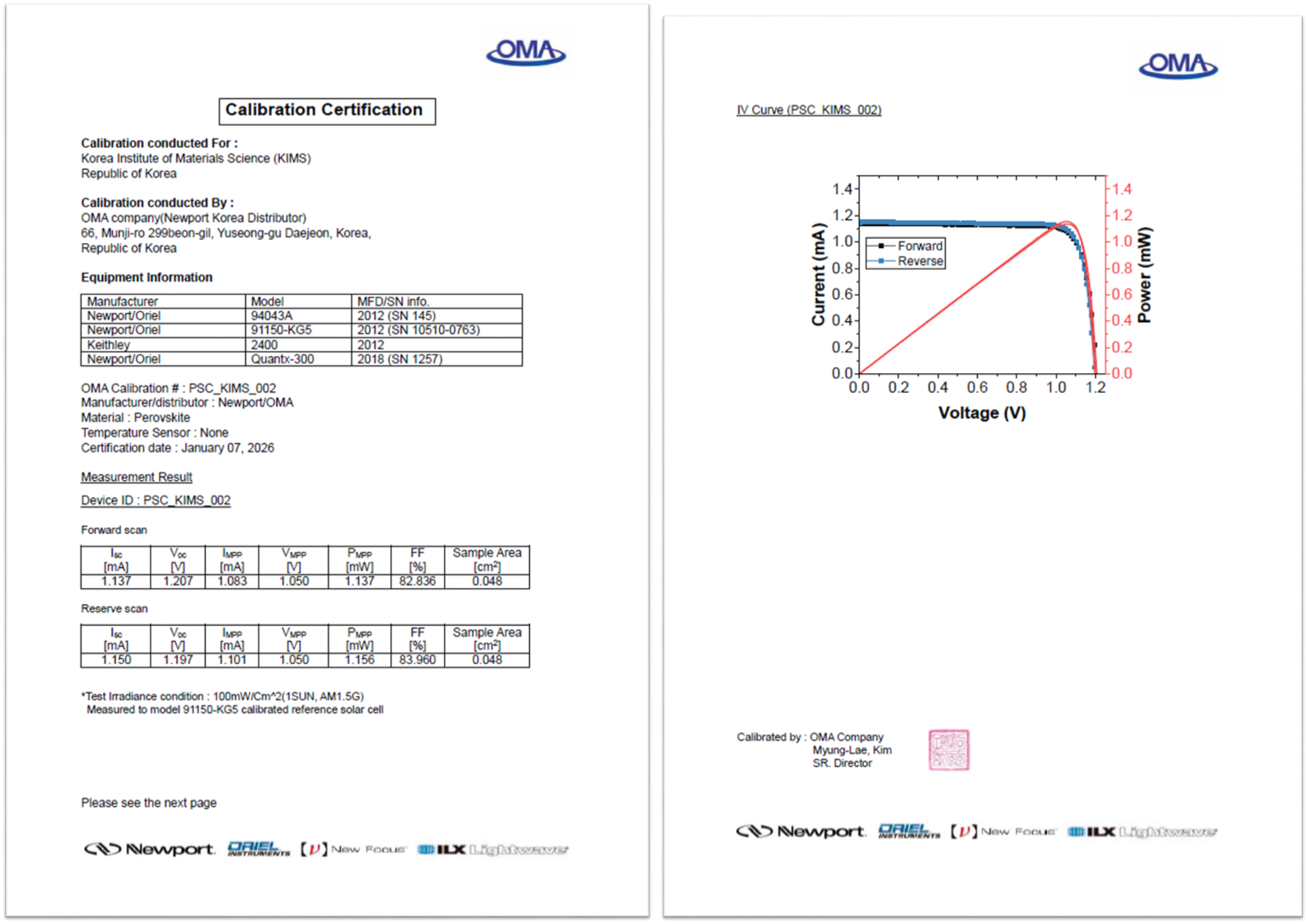


**Fig. S14** Certified PV performance for the 1.61-eV perovskite cells with 0.048 cm^2^ aperture area from an accredited photovoltaic certification laboratory (OMA company, Korea). The certificated efficiency is 24.07%.


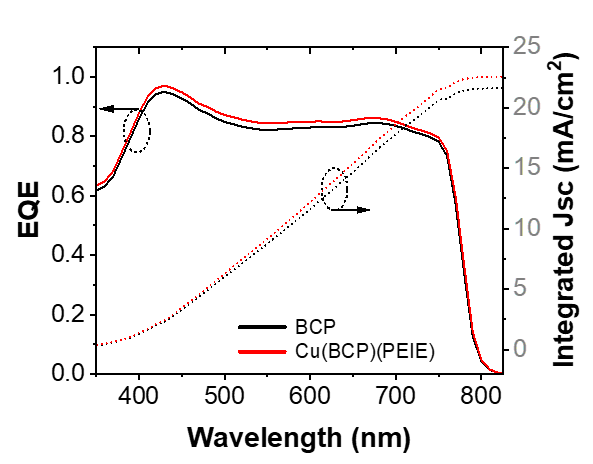


**Fig. S15** External quantum efficiency (EQE) and integrated *Jsc* value from the control (BCP) and target (Cu(BCP)(PEIE)) device.


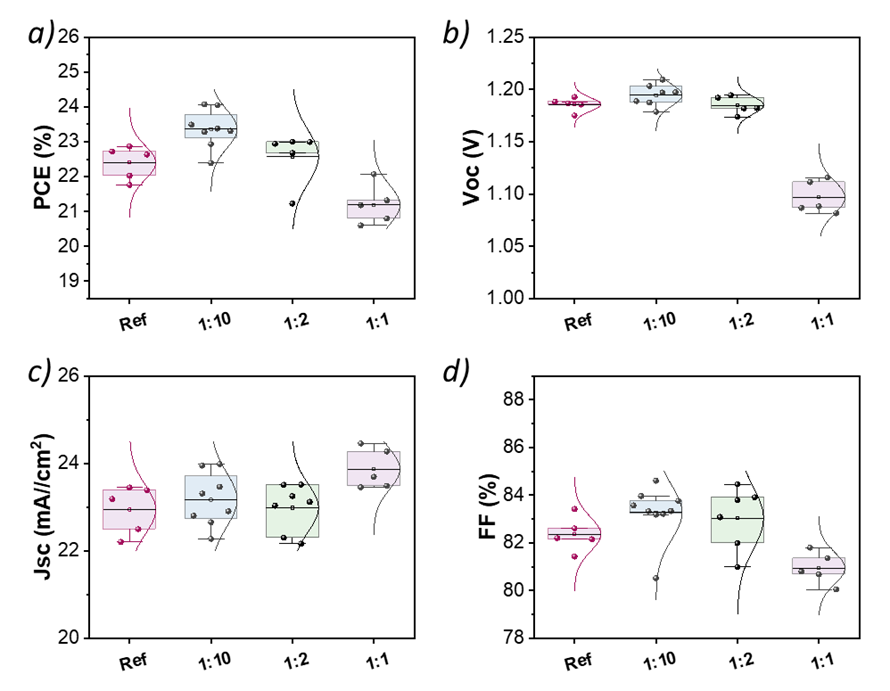


**Fig. S16** Device performance of PSCs with different CuCl_2_:BCP v/v ratio. (A) PCE, (B) *Voc,* (C) *Jsc*, and (D) *FF*.


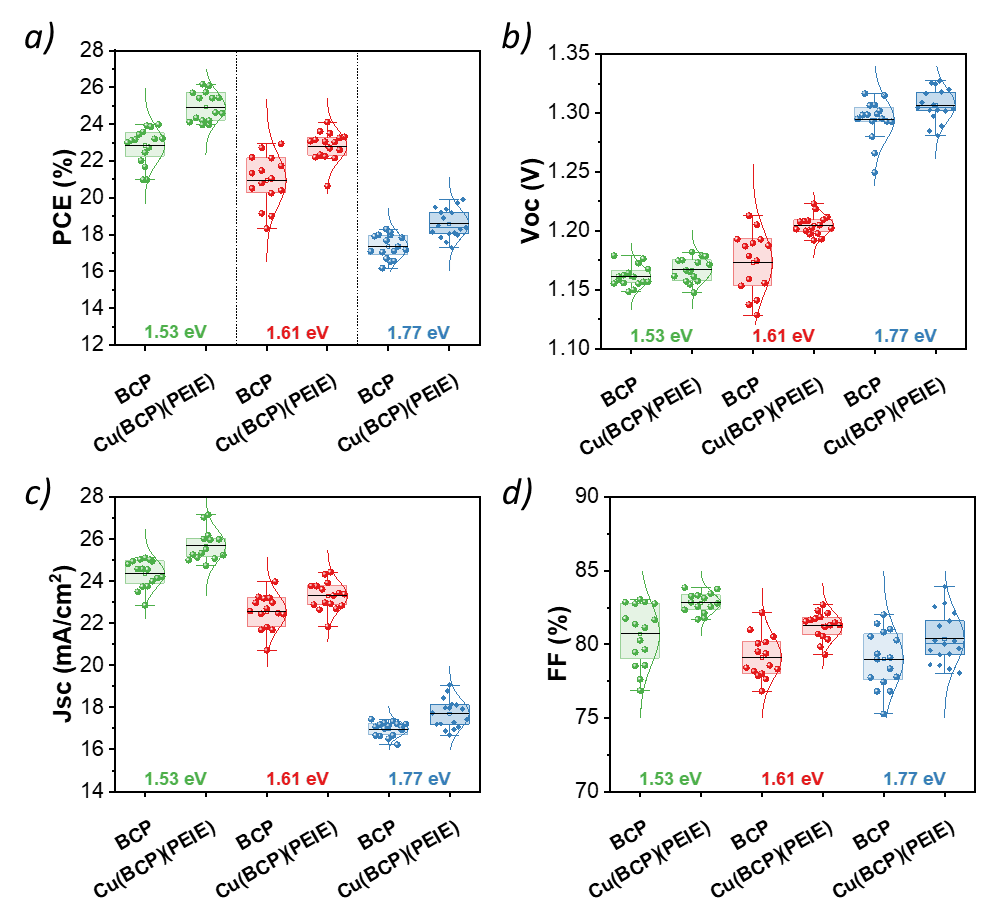


**Fig. S17** Performance metrics statistic of rigid PSCs with different bandgap. a) PCE, b) Voc, c) Jsc, and d) FF. Extracted from 15 different device.


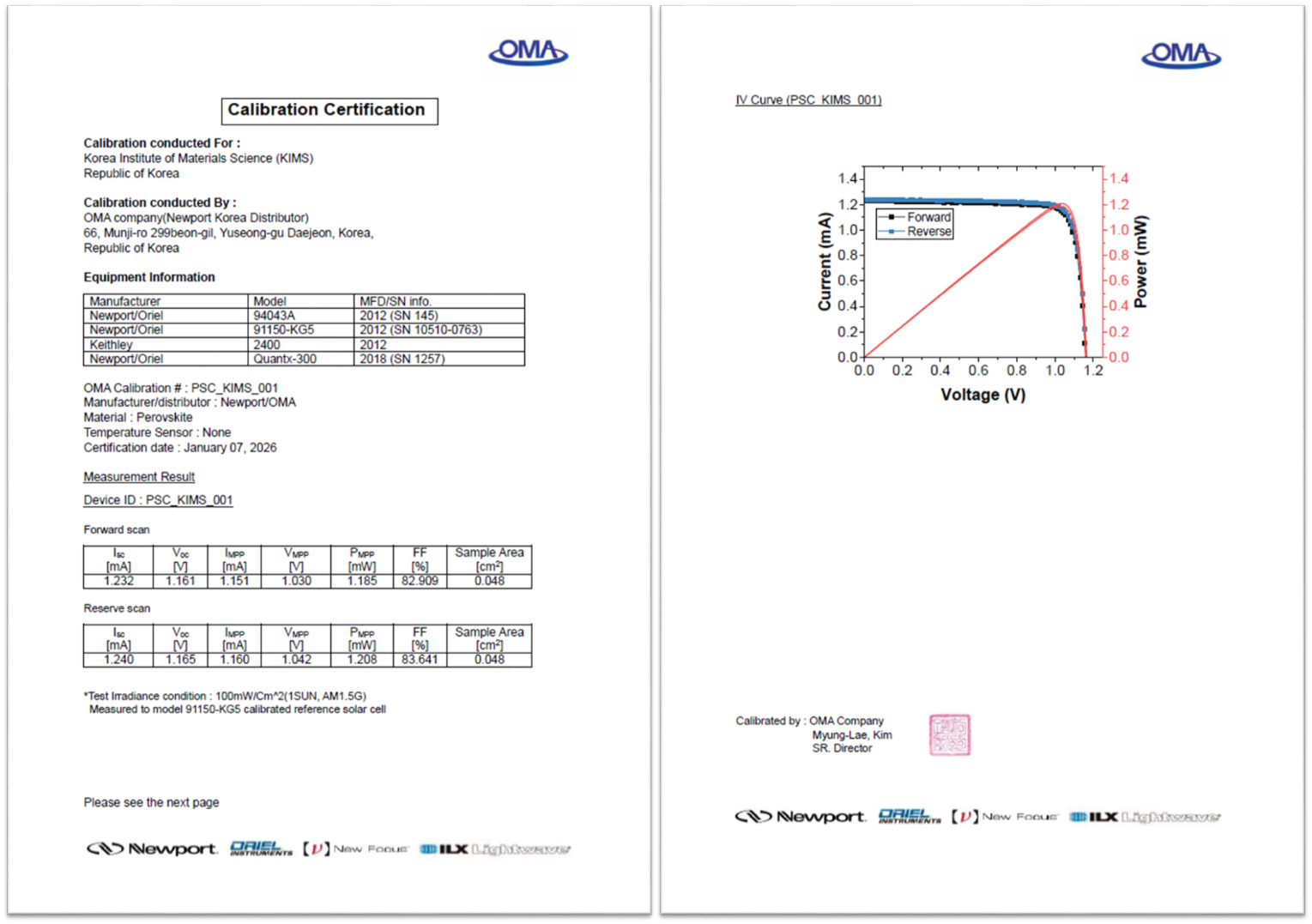


**Fig. S18** Certified PV performance for the 1.53-eV perovskite cells with 0.048 cm^2^ aperture area from an accredited photovoltaic certification laboratory (OMA company, Korea). The certificated efficiency is 25.18%.


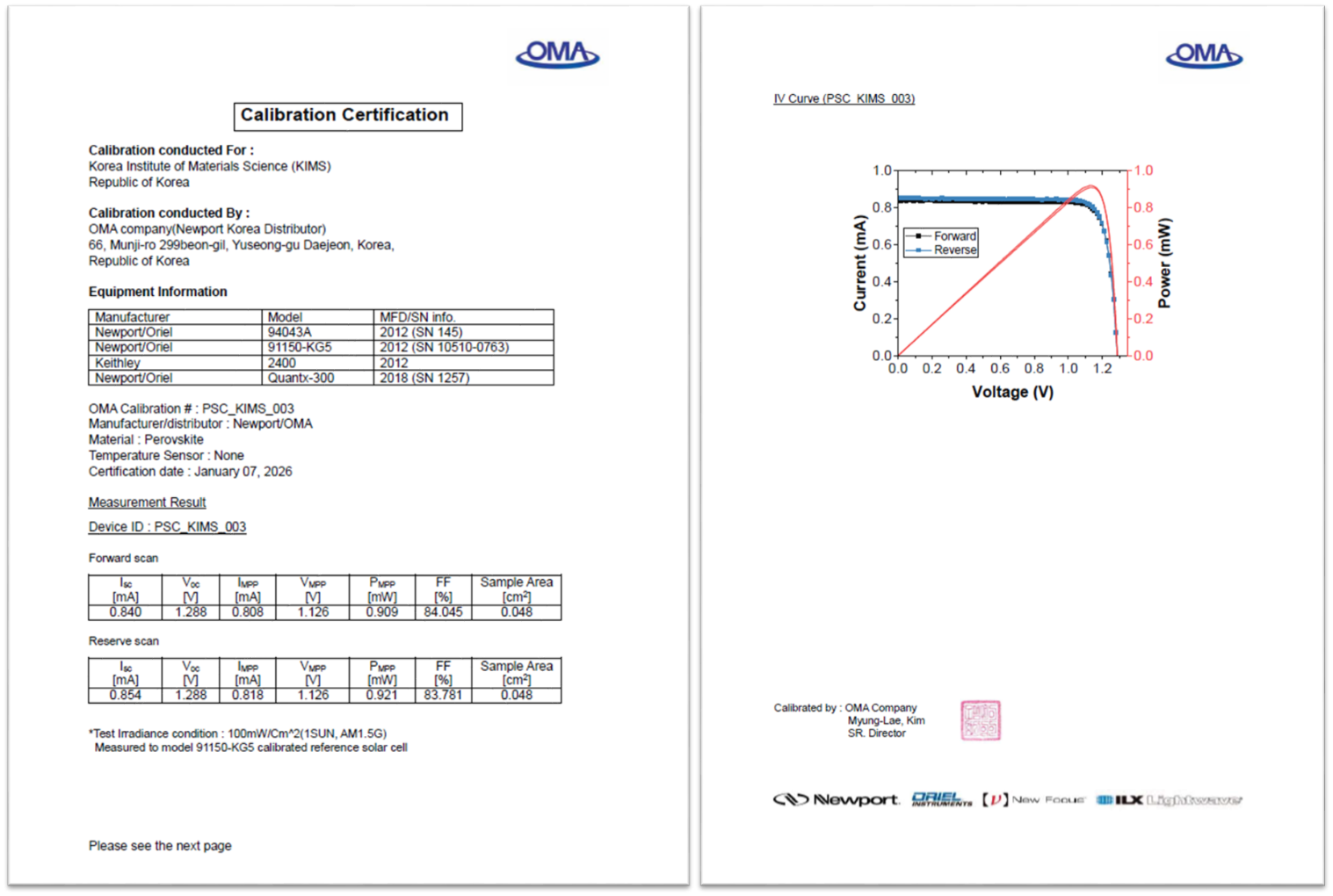


**Fig. S19** Certified PV performance for the 1.77-eV perovskite cells with 0.048 cm^2^ aperture area from an accredited photovoltaic certification laboratory (OMA company, Korea). The certificated efficiency is 19.19%.


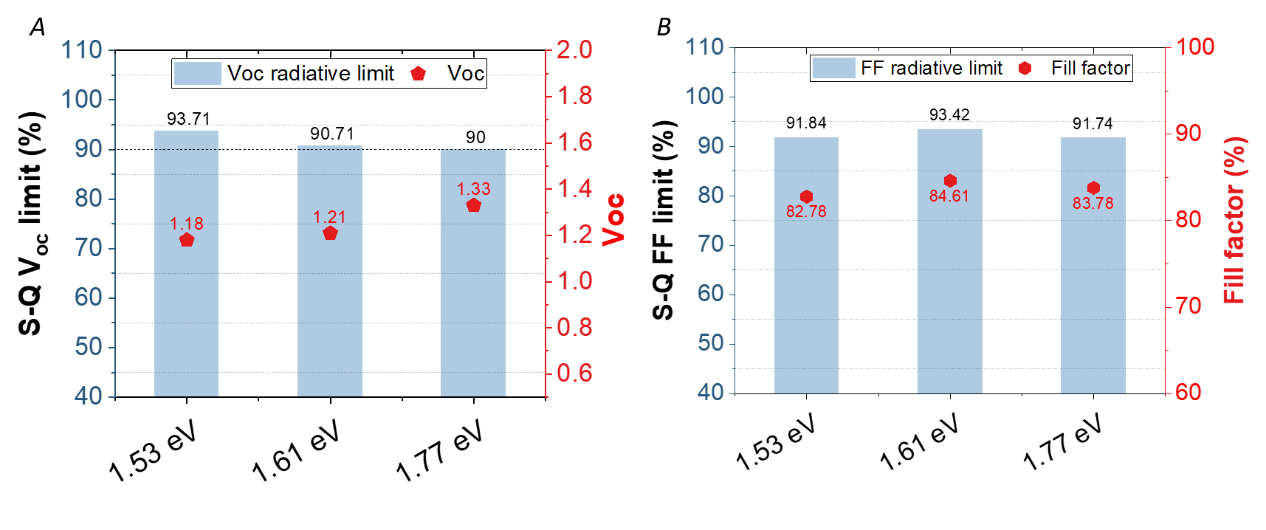


**Fig. S20** Percentage of the radiative limit and values of the champion device. (A) *V_oc_* and (b) *FF* for target devices with different bandgaps.


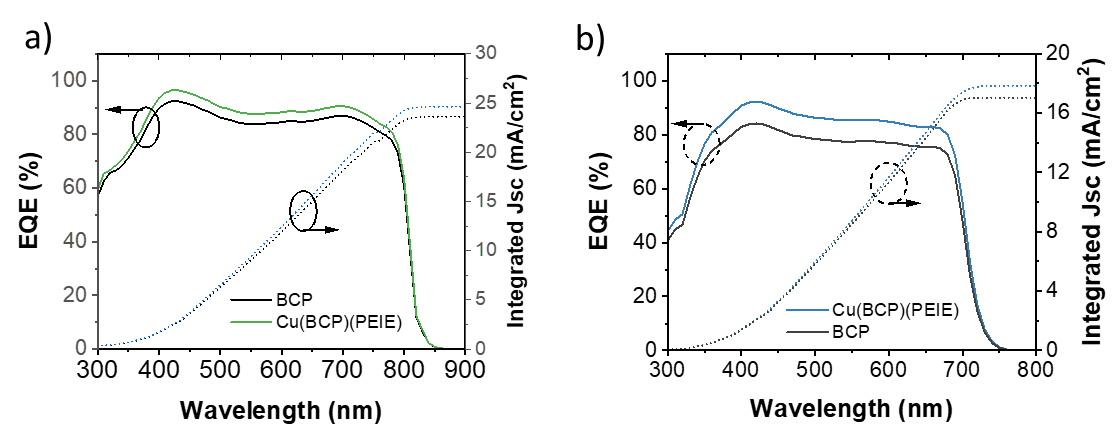


**Fig. S21** External quantum efficiency (EQE) and integrated Jsc value from the control (BCP) and target (Cu(BCP)(PEIE)) device for a) 1.53-eV bandgap perovskite and b) 1.77-eV bandgap perovskite


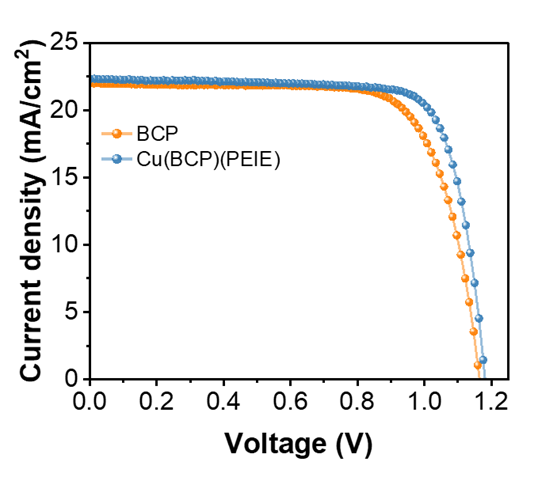


**Fig. S22** Device performance of air-processed (>25% RH) 1 cm^2^ area rigid PSCs with different buffer layer


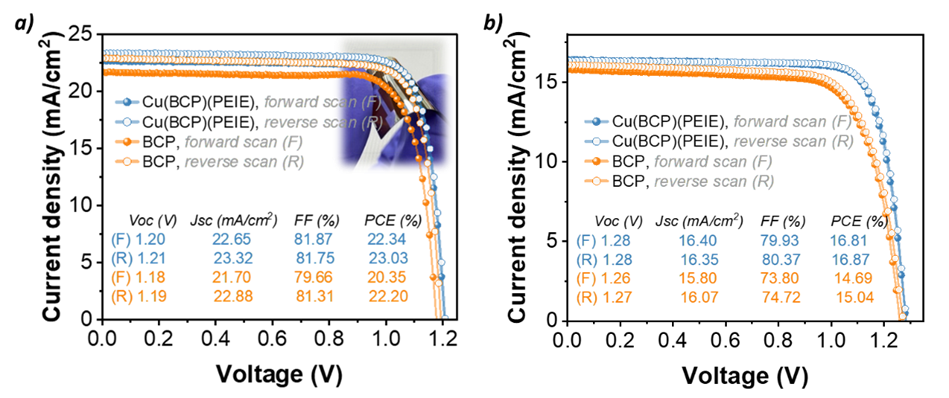


**Fig. S23** J–V characteristics of air-processed flexible perovskite solar cells (f-PSCs). (A) 1.61-eV perovskite. (B) 1.77-eV perovskite with BCP and Cu(BCP)(PEIE) buffer layers under AM 1.5G illumination, along with their corresponding performance parameters. The relative humidity during fabrication was over 45 %RH.


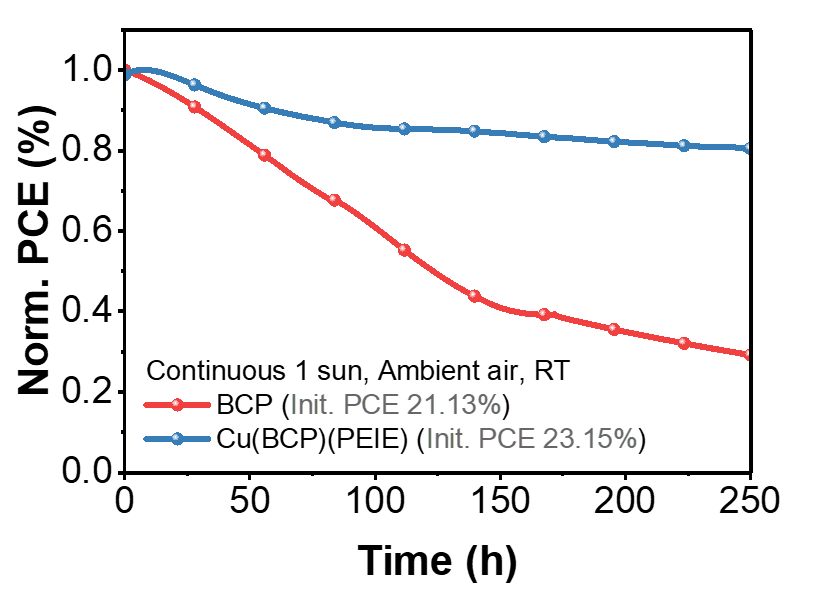


**Fig. S24** Maximum power point tracking (MPPT) test of BCP- and Cu(BCP)(PEIE)-based perovskite in ambient air, room temperature (RT) with continuous 1-sun illumination.


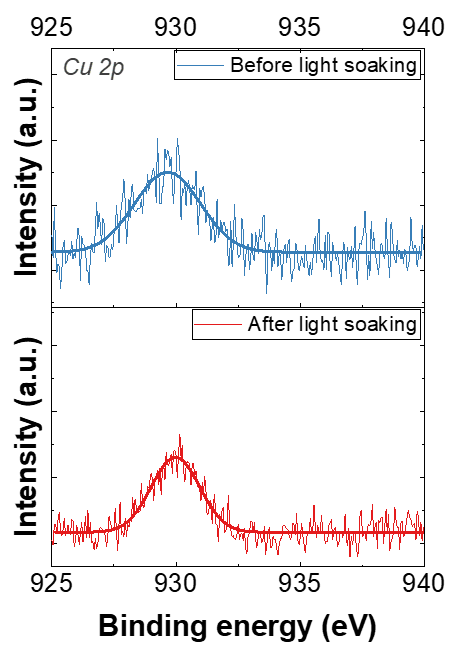


**Fig. S25** XPS Cu 2p spectrum of Cu(BCP)(PEIE) confirming the presence of Cu element before and after light-soaking for 200 h.


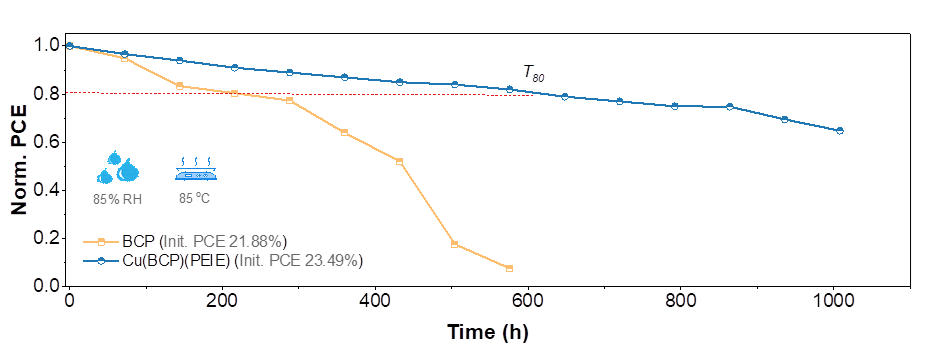


**Fig. S26** Thermal and moisture stability (85 ^o^C / 85 %RH) of encapsulated perovskite solar cells with different buffer layers.


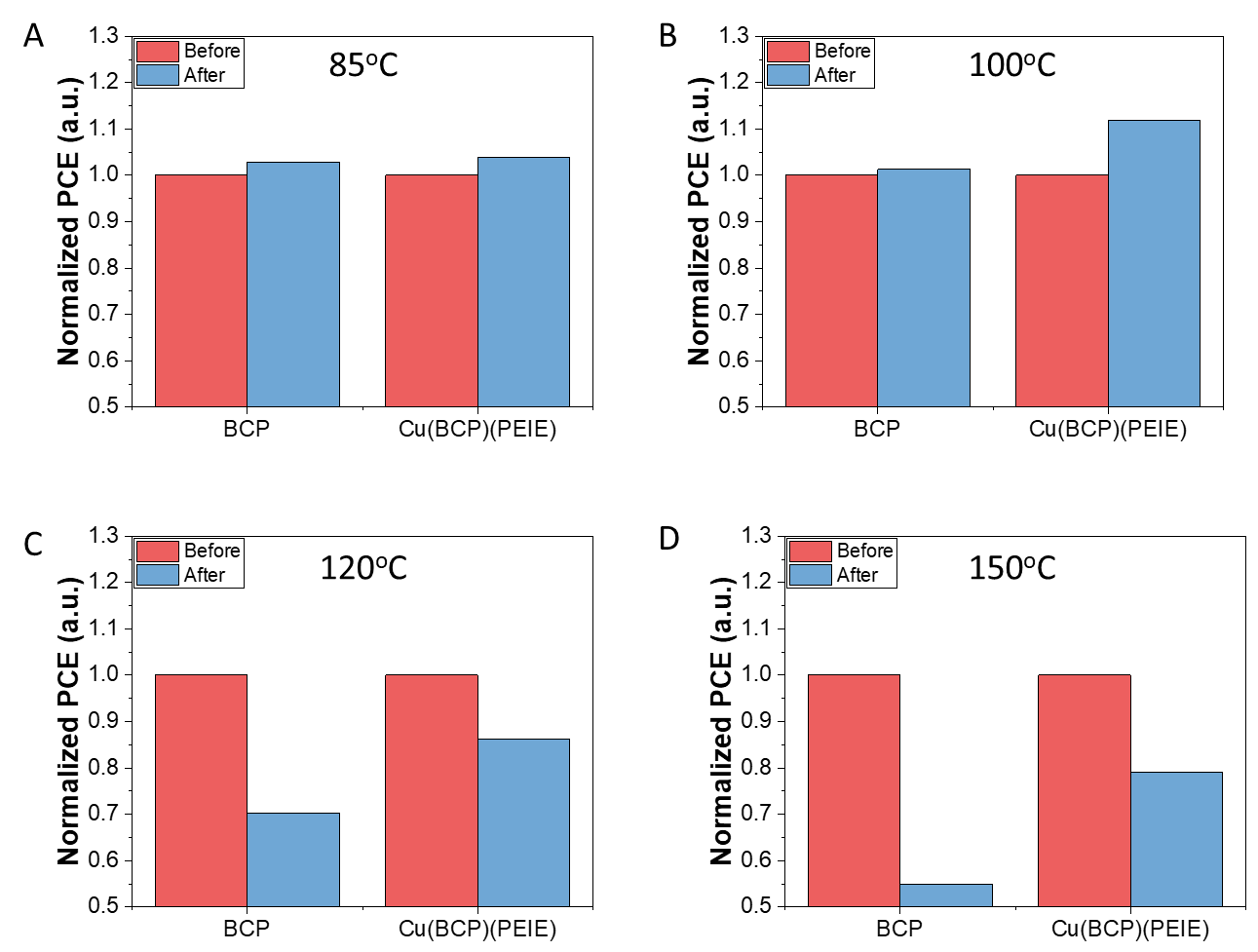


**Fig. S27** Post-thermal treatment of perovskite solar cells at different temperatures. (A) 85 ^o^C, (B) 100 ^o^C, (C) 120 ^o^C and (D) 150 ^o^C. Humidity during testing is ~55 % RH. Thermal treatment duration was 10 minutes.


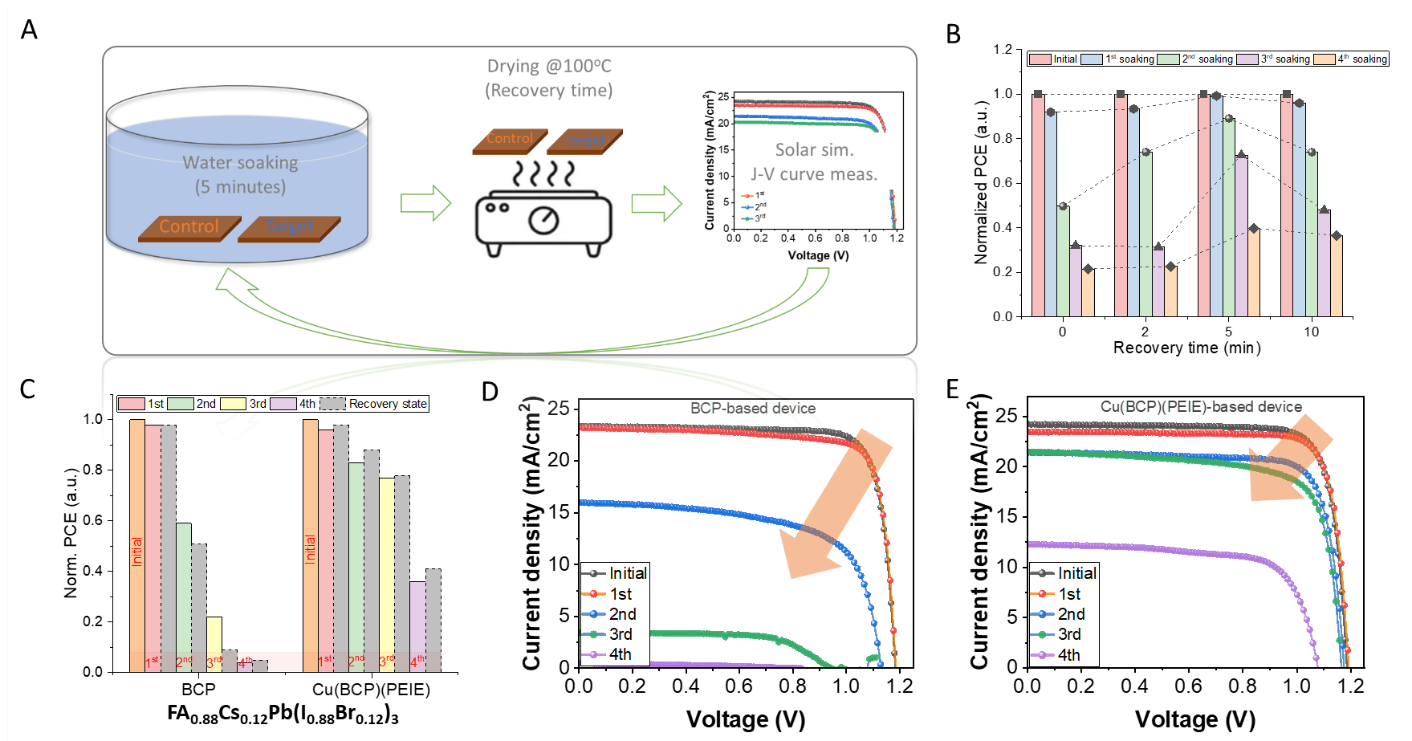


**Fig. S28** Water immersion test of perovskite solar cells. (A) a schematic representation of the water immersion test sequence and corresponding conditions. The water temperature during the test was kept at 22-25 ^o^C. (B) Normalized power conversion efficiency (PCE) of 1.61 eV perovskite solar cells after 5 min of water immersion followed by different durations of post-immersion thermal treatment. In this manuscript, this thermal-treatment period is referred to as the “restoration time”. (C) Normalized PCE evolution during repetitive water immersion tests of FA₀.₈₈Cs₀.₁₂Pb(I₀.₈₈Br₀.₁₂)₃ perovskite solar cells incorporating either the control (BCP) or target (Cu(BCP)(PEIE)) interlayer, together with the corresponding absolute PCE values measured after 5 min of recovery (post-immersion thermal treatment). The detailed photovoltaic parameters is summarized in Table S10-S11. After thermal treatment, the Cu(BCP)(PEIE)-based devices exhibit a markedly superior performance recovery compared with BCP-based control devices. This recovery behavior is attributed to the reversible hydration–dehydration process of the perovskite absorber and the enhanced moisture-blocking capability and interfacial defect passivation provided by the Cu(BCP)(PEIE) interlayer, which suppresses irreversible decomposition and facilitates efficient charge extraction after water exposure. (D-E) J-V curve evolution of recovered devices for BCP- and Cu(BCP)(PEIE)-based cells, respectively, after each # immersion cycles. Devices incorporating the target interlayer exhibit significantly improved resistance to water exposure compared with control devices, demonstrating enhanced interfacial robustness against moisture ingress.


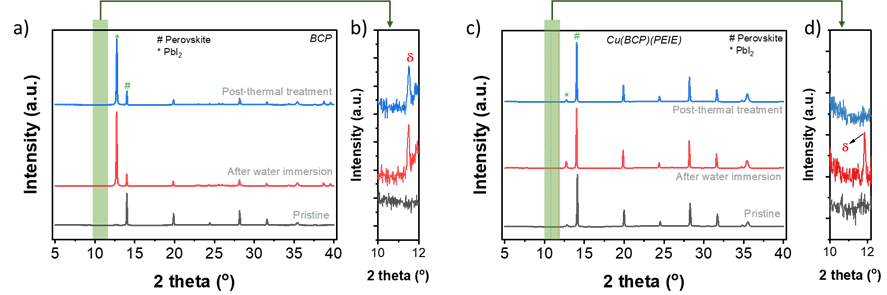


**Fig. S29** Crystallographic evidence of moisture-driven α-to-δ phase conversion and thermally assisted partial recovery in perovskite films across three stages (pristine, after water immersion, and after thermal treatment at 100 °C for 10 min). a) full-range XRD patterns of BCP-based films, b) enlarged 2θ region (10–12°) of BCP-based films confirming the non-perovskite δ-FAPbI₃ phase (δ), c) full-range XRD patterns of Cu(BCP)(PEIE)-based films, and d) enlarged 2θ region (10–12°) of Cu(BCP)(PEIE)-based films showing differential suppression of the δ-phase upon annealing.


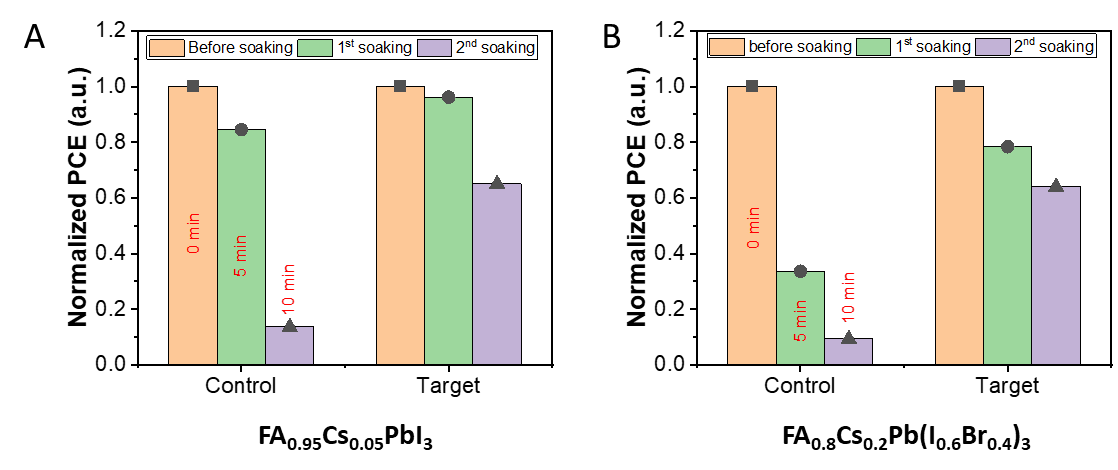


**Fig. S30** Normalized PCEs after 5 minutes of water immersion, followed by 5 min of thermal treatment (recovery) with different perovskite bandgap for both control (BCP) and target (Cu(BCP)(PEIE))-based cells. a) FA_0.95_Cs_0.05_PbI_3_ and b) FA_0.8_Cs_0.2_Pb(I_0.6_Br_0.4_)_3_.


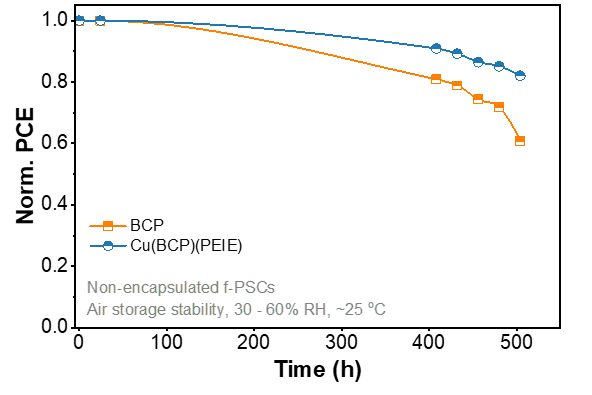


**Fig. S31** Air‑storage stability of non‑encapsulated flexible perovskite solar cells (f‑PSCs) employing BCP and Cu(BCP)(PEIE) showing the evolution of normalized PCE as a function of storage time under ambient conditions (30–60% relative humidity, ~25 ℃)


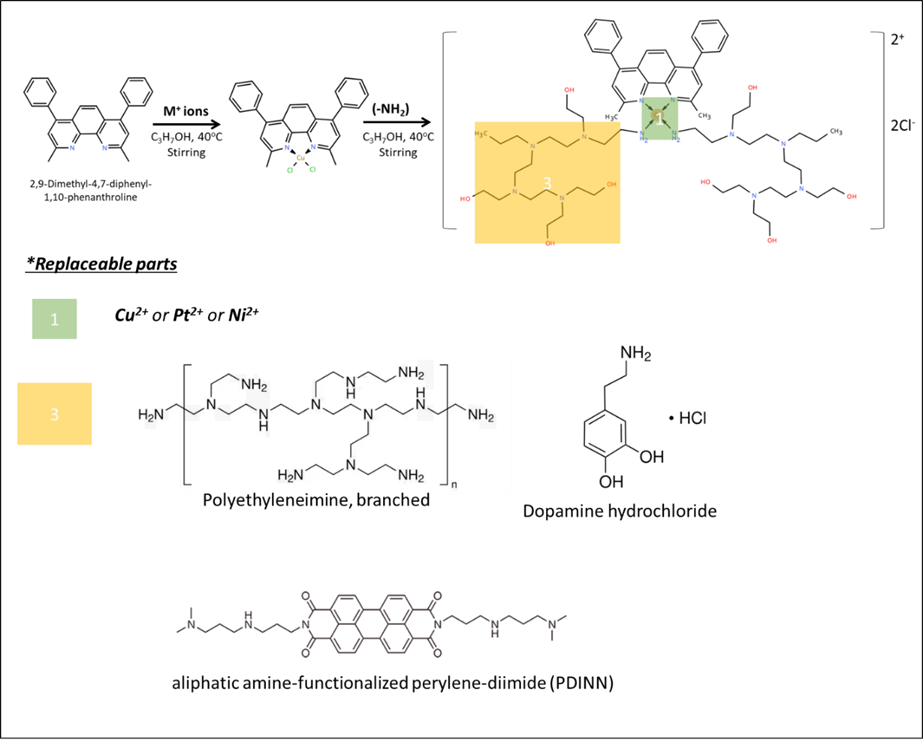


**Fig. S32** Illustration of replaceable parts in M(BCP)(-NH2-) molecular inter-net concept using various metal ions and amine-functionalized organic compound.


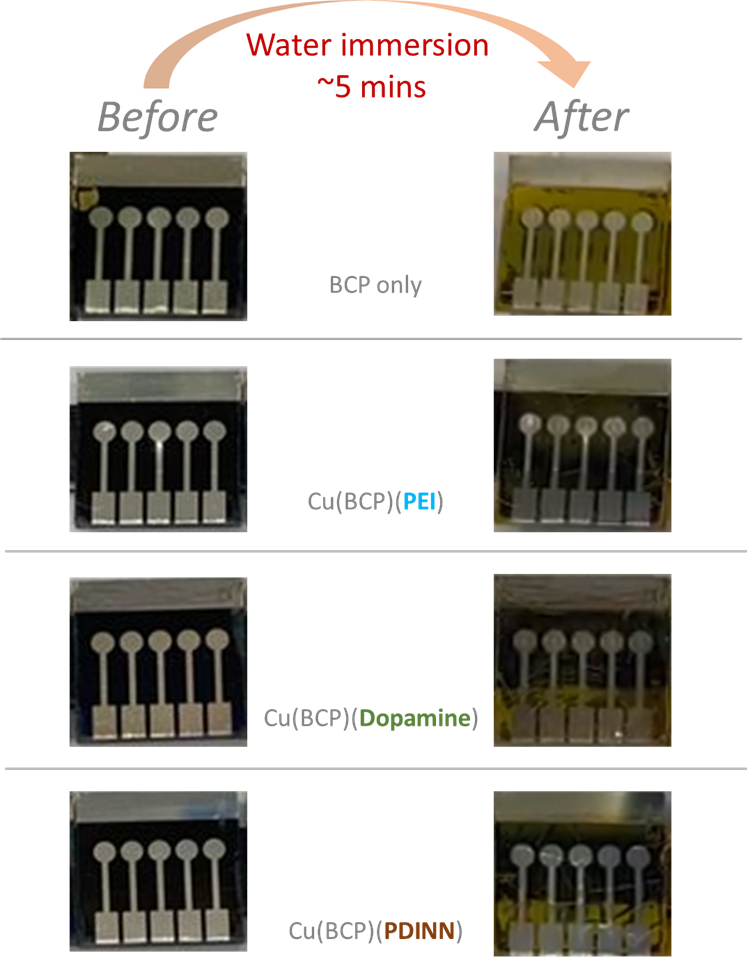


**Fig. S33** Water-immersion stability (5 minutes of dipping time) of inverted perovskite solar cells with different buffer layers.


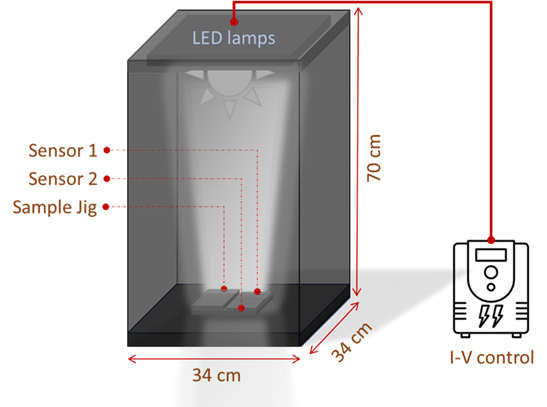


**Fig. S34** Indoor light measurement setup


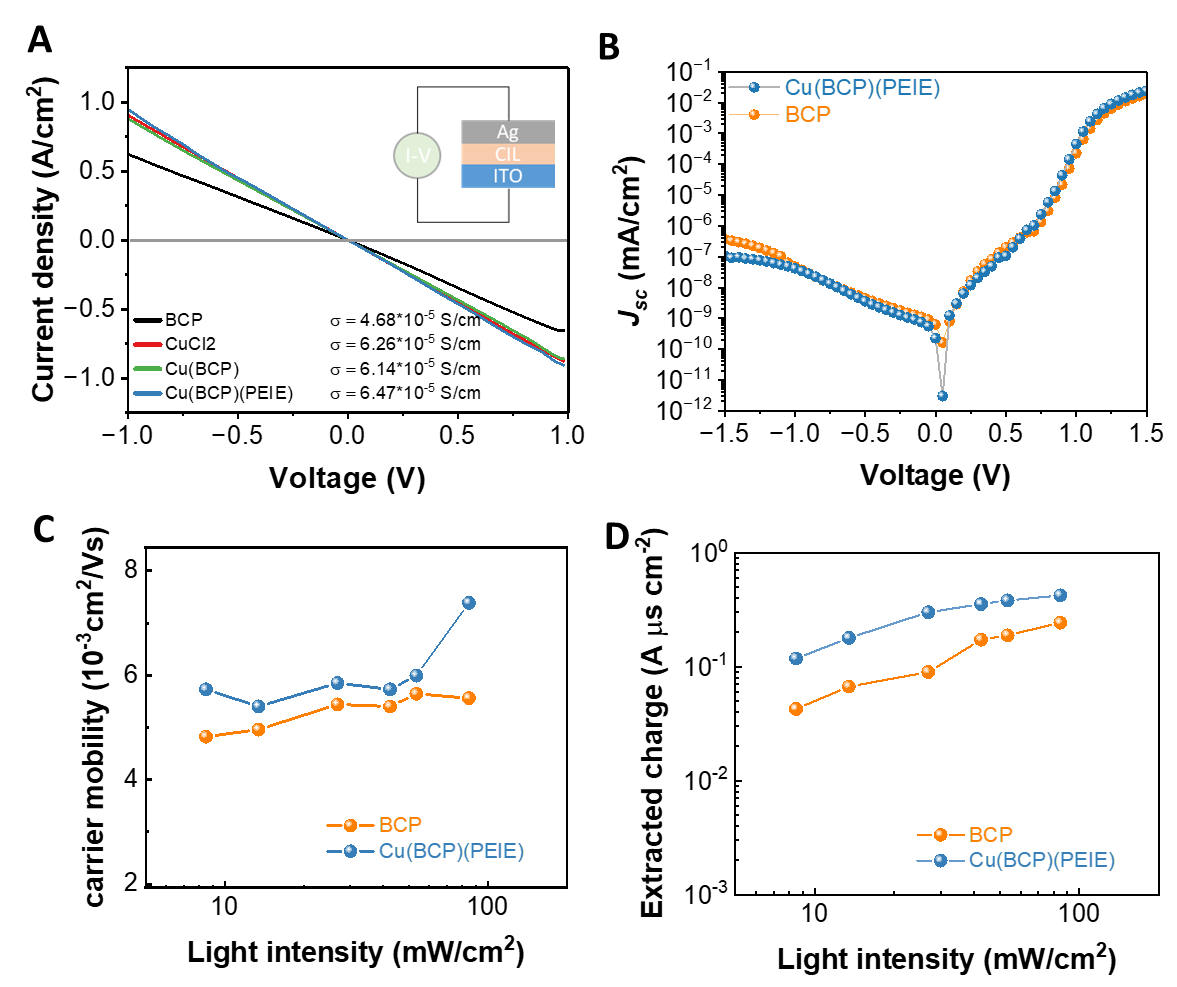


**Fig. S35** Electrical characterization and recombination analysis. a) I-V curves of different cathode interlayer materials. b) Measured J-V curve under dark conditions. c) Carrier mobility and d) extracted charge values (Qext) from light-dependent photo-CELIV curves.


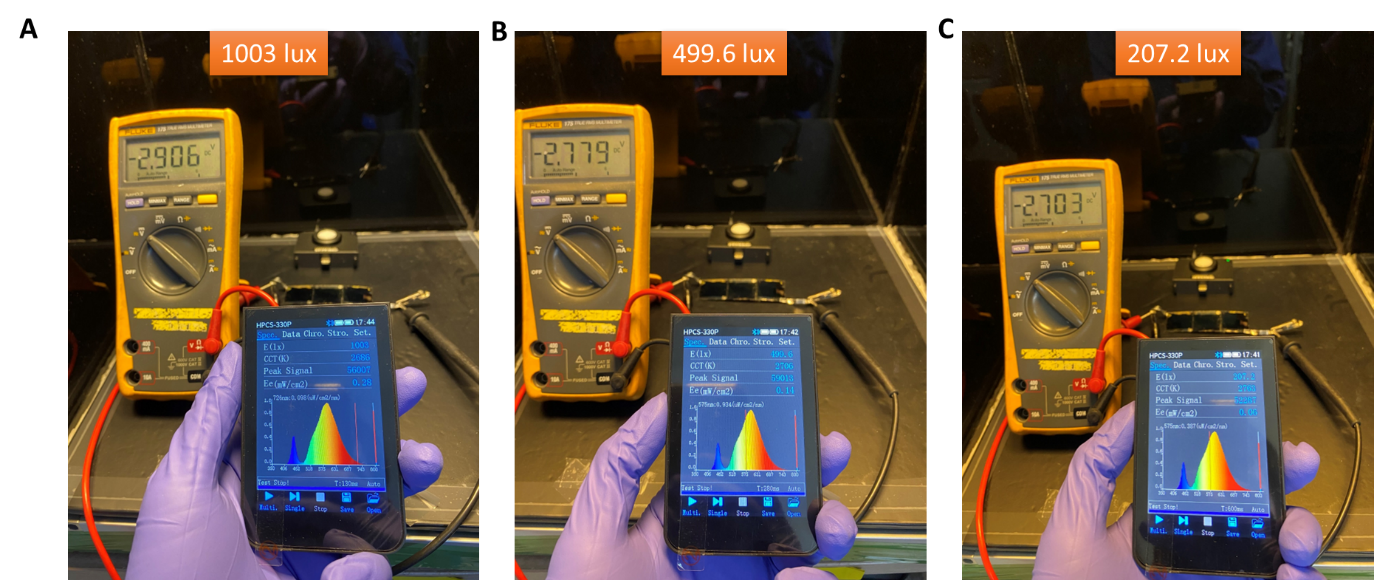


**Fig. S36** Voltage output generated from 3-connected devices under different low light intensities. a) 1003 lux, b) 499.6 lux, and c) 207.2 lux


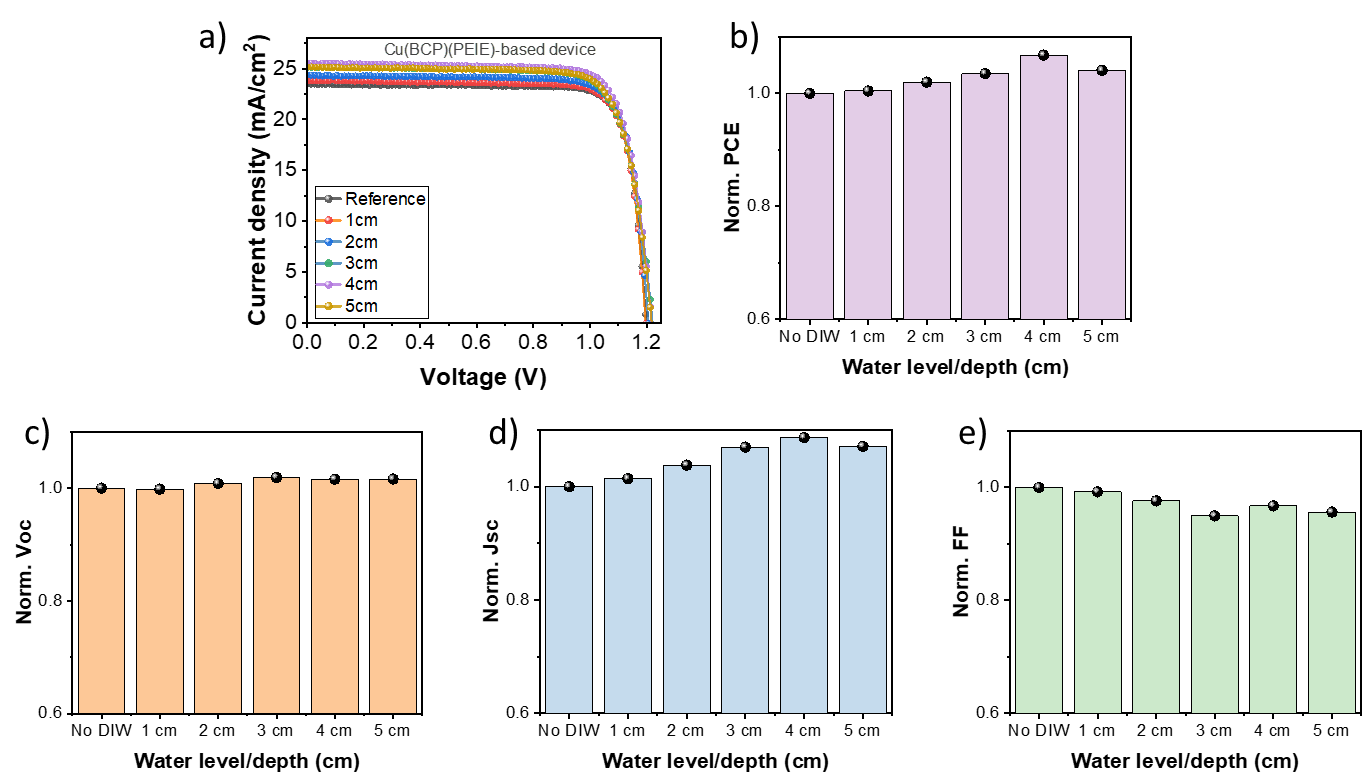


**Fig. S37** Water immersion test of perovskite solar cells with different water depth level. a) *J-V* curve, b) PCE, c) Voc, d) Jsc, and e) FF.

**Fig. S38** ICP-MS analysis of Pb^2+^ and Cs^+^ concentrations in water after immersion of perovskite solar cells with no buffer layer, BCP, and Cu(BCP)(PEIE) interlayers.

**Table S1** The integrated area and full-width at half maximum (FWHM) of Pb 4f peaks from XPS spectra with different cathode interlayer

| **Device** | **Aging temperature** | **FWHM** | | **Integrated Area** | |
| --- | --- | --- | --- | --- | --- |
|  |  | **Pb 4f_7/2_** | **Pb 4f_5/2_** | **Pb 4f_7/2_** | **Pb 4f_5/2_** |
| No buffer layer | RT | 1.21 | 1.24 | 8858 | 7250 |
|  | 100^o^C | 1.51 | 1.52 | 10874 | 8631 |
| BCP | RT | 1.50 | 1.57 | 25437 | 21304 |
|  | 100^o^C | - | - | - | - |
| Cu(BCP)(PEIE) | RT | 1.13 | 1.16 | 6398 | 4997 |
|  | 100^o^C | - | - | - | - |

**Table S2** The integrated area and full-width at half maximum (FWHM) of I 3d peaks from XPS spectra with different cathode interlayer

| **Device** | **Aging temperature** | **FWHM** | | **Integrated Area** | |
| --- | --- | --- | --- | --- | --- |
|  |  | I 3d_5/2_ | I 3d_3/2_ | I 3d_5/2_ | I 3d_3/2_ |
| No buffer layer | RT | 1.04 | 1.03 | 84985 | 54743 |
|  | 100^o^C | 1.30 | 1.33 | 93932 | 66255 |
| BCP | RT | 0.98 | 0.97 | 22651 | 15208 |
|  | 100^o^C | 1.02 | 0.97 | 63819 | 39050 |
| Cu(BCP)(PEIE) | RT | 0.95 | 0.89 | 30282 | 18655 |
|  | 100^o^C | 1.02 | 1.06 | 64178 | 46217 |

**Table S3** Comparison of photovoltaic performance of representative perovskite solar cells with bandgaps >1.6 eV reported in the literature and in this work

| **Bandgap**  **(eV)** | **Voc**  **(V)** | **Jsc**  **(mA/cm^2^)** | **Fill factor**  **(%)** | **PCE**  **(%)** | **References** |
| --- | --- | --- | --- | --- | --- |
| 1.6 | 1.22 | 22.3 | 83.4 | 22.6 | *Science*2024, 384, 767. |
| 1.61 | 1.164 | 23.19 | 85.7 | 23.13 | *InfoMat.* 2023;5:e12379 |
| **1.61** | **1.21** | **23.51** | **84.61** | **24.11** | ***This work*** |
| 1.65 | 1.206 | 22.42 | 81.55 | 22.05 | *Nat. Energy*2024, 9, 536. |
| 1.66 | 1.241 | 20.89 | 83.89 | 21.62 | *Nano Energy*2024, 121, 109162. |
| 1.67 | 1.26 | 19.5 | 84 | 20.6 | *Science*2024, 384, 767. |
| 1.68 | 1.282 | 22.43 | 81.2 | 23.35 | *Energy Environ. Sci.*2024, 17, 5866. |
| 1.7 | 1.26 | 20.84 | 82.82 | 21.85 | *Energy Environ. Sci.*2025, 18, 1232. |
| 1.72 | 1.321 | 19.32 | 76.1 | 19.42 | *Nat. Commun.*2024, 15, 882. |
| 1.73 | 1.27 | 19.15 | 81.89 | 19.97 | *Science*2024, 383, 524. |
| 1.75 | 1.32 | 18.81 | 83.59 | 20.8 | *Nat. Commun.*2024, 15, 8899. |
| 1.77 | 1.36 | 18.3 | 82.3 | 20.5 | *Nature*2024, 635, 867. |
| **1.77** | **1.32** | **18.73** | **81.46** | **20.00** | ***This work*** |
| 1.78 | 1.311 | 18.3 | 83.1 | 19.9 | *Joule*2025, 101801. |
| 1.79 | 1.332 | 17.37 | 82.9 | 19.59 | *Nat. Energy*2024, 9, 298. |
| 1.8 | 1.274 | 17.4 | 79.6 | 17.6 | *Chem. Eng. J.*2024, 482, 148638. |
| 1.81 | 1.351 | 17.52 | 82.74 | 19.58 | *Nat. Energy*2024, 9, 411. |
| 1.82 | 1.305 | 17.36 | 84.82 | 19.22 | *Adv. Energy Mater.*2024, 2404366. |
| 1.83 | 1.32 | 17.06 | 84.21 | 18.96 | *Nat. Energy*2024, 9, 592. |
| 1.85 | 1.258 | 14.3 | 71.8 | 12.9 | *ACS Energy Lett.*2024, 9, 1666. |
| 1.86 | 1.366 | 16.1 | 84.2 | 18.52 | *Joule*2024, 8, 2554. |
| 1.88 | 1.36 | 16.1 | 83.8 | 18.1 | *Nature*2024, 635, 860. |
| 1.91 | 1.31 | 14.78 | 77.9 | 15.25 | *Nat. Energy*2023, 9, 70. |
| 1.93 | 1.422 | 14.18 | 93.79 | 16.9 | *Nature*2024, 628, 306. |
| 1.96 | 1.33 | 13.05 | 76.7 | 13.4 | *Adv. Mater.*2024, 36, 2311595. |
| 1.97 | 1.44 | 12.8 | 0.83 | 15.3 | *Nat. Energy*2023, 9, 70. |
| 1.99 | 1.17 | 11.13 | 66.3 | 8.63 | *Angew. Chem., Int. Ed.*2024, 64, *202415966.* |
| 2.1 | 1.316 | 11.79 | 73.6 | 11.42 | *Nat. Energy*2023, 9, 70. |

**Table S4** Device performance of perovskite solar cells with different buffer layers

| **Condition** | **Voc**  **(V)** | **Jsc**  **(mA/cm^2^)** | **FF**  **(%)** | **PCE**  **(%)** |
| --- | --- | --- | --- | --- |
| BCP | 1.19 | 23.18 | 82.15 | 22.72 |
| CuCl2 | 1.05 | 20.99 | 54.04 | 11.88 |
| Cu(BCP) | 1.17 | 23.82 | 80.86 | 22.54 |
| Cu(BCP)(PEIE) | 1.21 | 23.51 | 84.61 | 24.11 |

**Table S5** Different Cu:BCP ratio effect in device performance

| **Condition** | **Voc**  **(V)** | **Jsc**  **(mA/cm^2^)** | **FF**  **(%)** | **PCE**  **(%)** |
| --- | --- | --- | --- | --- |
| Control (BCP only) | 1.19 | 23.18 | 82.15 | 22.72 |
| Cu:BCP  1:10 v/v ratio | 1.21 | 23.51 | 84.61 | 24.11 |
| 1:2 v/v ratio | 1.17 | 22.91 | 83.22 | 22.39 |
| 1:1 v/v ratio | 1.12 | 23.13 | 76.38 | 19.77 |

**Table S6** Device performance of perovskite solar cells with BCP and different PEIE concentrations

| **Condition** | **Voc**  **(V)** | **Jsc**  **(mA/cm^2^)** | **FF**  **(%)** | **PCE**  **(%)** |
| --- | --- | --- | --- | --- |
| Control (BCP only). ~6 nm | 1.17 | 23.34 | 81.86 | 22.36 |
| Cu(BCP)(0.01 wt% PEIE). ~7 nm | 1.18 | 22.27 | 83.34 | 21.88 |
| Cu(BCP)(0.025 wt% PEIE). ~9 nm | 1.20 | 23.31 | 83.76 | 23.49 |
| Cu(BCP)(0.05 wt% PEIE). ≥14 nm | 1.19 | 23.98 | 80.52 | 22.93 |

**Table S7** Device performance of perovskite solar cells with BCP and different PEIE concentrations

| **Cu(BCP)(PEIE) coating speed** | **Voc**  **(V)** | **Jsc**  **(mA/cm^2^)** | **FF**  **(%)** | **PCE**  **(%)** |
| --- | --- | --- | --- | --- |
| 2000 rpm. ~11 nm | 1.21 | 23.62 | 80.71 | 23.05 |
| 4000 rpm. ~9 nm | 1.21 | 23.92 | 81.30 | 23.50 |
| 6000 rpm. ~6 nm | 1.17 | 23.59 | 78.99 | 21.86 |

**Table S8** Device performance of 0.048 cm^2^ and 1 cm^2^ rigid perovskite solar cells (1.61 eV bandgap)

| **Condition** | **Active area (cm^2^)** | **Voc**  **(V)** | **Jsc**  **(mA/cm^2^)** | **FF**  **(%)** | **PCE**  **(%)** | **Ratio of PCE loss cells to 1cm^2^** |
| --- | --- | --- | --- | --- | --- | --- |
| Control (BCP only) | 0.048 (small) | 1.17 | 23.34 | 81.86 | 22.72 | ~15.7% |
|  | 1 (large) | 1.17 | 22.29 | 73.45 | 19.15 |  |
| Cu(BCP)(PEIE) | 0.048 (small) | 1.20 | 23.95 | 83.96 | 24.07 | ~7.8% |
|  | 1 (large) | 1.19 | 22.88 | 81.31 | 22.20 |  |

**Table S9** Device performance of air-processed large area (1 cm^2^) rigid perovskite solar cells (RH >45%)

| **Condition** | **Scan direction** | **Voc**  **(V)** | **Jsc**  **(mA/cm^2^)** | **FF**  **(%)** | **PCE**  **(%)** |
| --- | --- | --- | --- | --- | --- |
| Control (BCP only) | *Forward* | 1.18 | 22.80 | 75.02 | 18.57 |
|  | *Reverse* | 1.16 | 21.97 | 73.43 | 18.80 |
| Cu(BCP)(PEIE) | *Forward* | 1.19 | 22.71 | 73.82 | 20.02 |
|  | *Reverse* | 1.18 | 22.29 | 77.56 | 20.42 |

**Table S10** Comparison of photovoltaic performance parameters of air-processed flexible PSCs

| **Device architecture** | **Active area**  **(cm^2^)** | **Bandgap**  **(eV)** | **Fabrication**  **conditions** | **FF**  **(%)** | **PCE (%)** | **Refs.** |
| --- | --- | --- | --- | --- | --- | --- |
| PET/ITO/SnO_2_/perovskite/  spiro-OMeTAD/Au | 0.09 | 1.55 | Ambient air | 79.9 | 23.01 | *Energy Environ. Sci.* 2024, 17, 7069-7080. |
| PET/ITO/MeO-2PACz: PEACl/  perovskite/PEACl/PCBM/BCP/Ag | 0.14 | 1.61 | Ambient air | 72.11 | 19.20 | *Chem. Eng. J.* 2025, 518, 164371. |
| PEN/ITO/SnO_2_/perovskite/PEDOT: PSS/carbon | 0.062 | 1.55 | Ambient air | 80 | 19.70 | *Adv. Funct. Mater.* 2024, 34, 2406354. |
| PET/ITO/SnO_2_/perovskite+AES/  spiro-OMeTAD/Au | 0.05 | 1.53 | 30-40% RH | 76 | 20.1 | *Small* 2024, 20, 2401456. |
| PET/ITO/PEDOT:PSS/perovskite+PDLLA/PCBM/BCP/Ag | 0.06 | 1.55 | 20-25 °C  <40 % RH | 74.5 | 16.61 | *J. Mater. Sci.* 2022, 57, 20654-20671. |
| PET/ITO/SnO_2_-PTACl/  perovskite/spiro-OMeTAD/Au | 1 | 1.55 | Ambient air | 80 | 17.6 | *Sol. RRL* 2024, 8, 2301013. |
| PET/ITO/SnO_2_/perovskite/spiro-OMeTAD/Au | 0.09 | 1.53 | 25% RH | 77.2 | 19.38 | *Adv. Funct. Mater.* 2019, 29, 1902974. |
| PEN/ITO/SnO_2_/perovskite+chitin/  spiro-OMeTAD/Au | 0.09 | 1.55 | 15-25% RH | 78.73 | 22.84 | *Adv. Mater.* 2025, 37, 2411982. |
| PEN/ITO/SnO_2_-DAC-AA/perovskite  /spiro-OMeTAD/MoO_x_/Ag | 0.092 | 1.54-1.55 | 20-25 °C, 30-40% RH | 81.45 | 23.87 | *Adv. Mater.* 2025, 37, 2418791. |
| PET/ITO/NiOx/MeO-4PACz/Perovskite/PEAI/PCBM/PEIE-CuBCP/BCP | **0.048** | **1.61** | **18-22 °C,**  **>45% RH** | **81.75** | **23.03** | **This work** |

**Table S11** The photovoltaics parameters evolution of the BCP-based device after #cycle of water immersion and corresponding results after thermal treatment after immersion (recovery period)

| **Water immersion cycling** | **Thermal-treatment (TA)**  **(5 min @100^o^C)** | **Voc**  **(V)** | **Jsc**  **(mA/cm^2^)** | **FF**  **(FF)** | **PCE**  **(%)** | **Retained PCE**  **(%)** |
| --- | --- | --- | --- | --- | --- | --- |
| Initial | - | 1.18 | 23.30 | 81.78 | 22.53 | 100 |
| 1^st^ soaking | before | 1.18 | 23.05 | 81.19 | 22.08 | 98.4 |
|  | After TA | 1.18 | 23.20 | 80.60 | 22.15 | 98.9 |
| 2^nd^ soaking | before | 1.10 | 16.32 | 74.17 | 13.3 | 59 |
|  | After TA | 1.13 | 15.95 | 64.73 | 11.66 | 51.8 |
| 3^rd^ soaking | before | 1.00 | 9.63 | 53.45 | 5.13 | 22.8 |
|  | After TA | 0.94 | 3.43 | 65.29 | 2.11 | 9.4 |
| 4^th^ soaking | before | 0.18 | 13.27 | 44.67 | 1.08 | 4.8 |
|  | After TA | 0.77 | 0.46 | 32.90 | 0.12 | 0.5 |

**Table S12** The photovoltaics parameters evolution of Cu(BCP)(PEIE)-based device after #cycle of water immersion and corresponding results after thermal treatment after immersion (recovery period)

| **Water immersion cycling** | **Thermal-treatment (TA)**  **(5 min @100^o^C)** | **Voc**  **(V)** | **Jsc**  **(mA/cm^2^)** | **FF**  **(FF)** | **PCE**  **(%)** | **Retained PCE**  **(%)** |
| --- | --- | --- | --- | --- | --- | --- |
| Initial | - | 1.19 | 24.22 | 81.56 | 23.4 | 100 |
| 1^st^ soaking | before | 1.18 | 23.58 | 81.35 | 22.58 | 96.5 |
|  | After TA | 1.19 | 23.45 | 82.62 | 23.09 | 98.7 |
| 2^nd^ soaking | before | 1.18 | 20.23 | 81.57 | 19.48 | 83.2 |
|  | After TA | 1.17 | 21.40 | 79.68 | 20.03 | 88.7 |
| 3^rd^ soaking | before | 1.17 | 21.47 | 72.23 | 18.18 | 77.7 |
|  | After TA | 1.16 | 21.46 | 73.91 | 18.47 | 78.9 |
| 4^th^ soaking | before | 1.07 | 14.75 | 54.10 | 8.57 | 36.6 |
|  | After TA | 1.08 | 12.27 | 70.43 | 9.31 | 39.8 |

**Table S13** Comparison of indoor performance for air-processed PSCs (1.61 eV perovskite)

| Intensity (Lux) | Device | V_OC_  (V) | J_SC_  (μA/cm^2^) | FF  (%) | PCE  (%) |
| --- | --- | --- | --- | --- | --- |
| 1000 | BCP | 0.97 | 113.83 | 82.10 | 32.84 |
|  | Cu(BCP)(PEIE) | 0.97 | 123.05 | 84.05 | 36.00 |
| 500 | BCP | 0.94 | 57.35 | 82.56 | 31.96 |
|  | Cu(BCP)(PEIE) | 0.96 | 61.13 | 83.21 | 35.40 |
| 200 | BCP | 0.92 | 22.30 | 80.69 | 30.02 |
|  | Cu(BCP)(PEIE) | 0.93 | 25.36 | 83.37 | 35.02 |

**Table S14** Reported low indoor light performance (≤ 500 lux intensities) of perovskite solar cells

| **Year** | **Color**  **temperature** | **Lux** | **Power input**  **(uW/cm^2^)** | **Light**  **source** | **structure** | **Voc**  **(V)** | **Jsc**  **(uA/cm^2^)** | **FF**  **(%)** | **PCE**  **(%)** | **Ref** |
| --- | --- | --- | --- | --- | --- | --- | --- | --- | --- | --- |
| 2025 | 2700K | 500 | 137.95 | Warm LED light | P-I-N | 0.96 | 61.13 | 83.21 | 35.40 | **This work** |
| 2025 | 2700K | 200 | 56.14 | Warm LED light | P-I-N | 0.93 | 25.36 | 83.37 | 35.02 | **This work** |
| 2025 | 4000K | 200 |  | White LED | N-I-P | 0.86 | 21.7 | 74.48 | 23.1 | Solar RRL, 2025; 9:e2500195 |
| 2024 | 4000K | 400 | 98.8 | WLED | P-I-N | 1.07 | 49.1 | 81.8 | 39.6 | Small 2025, 21, 2408271 |
| 2024 | 4000K | 200 | 49.5 | WLED | P-I-N | 1.06 | 25.1 | 81.9 | 39.7 | Small 2025, 21, 2408271 |
| 2023 |  | 400 |  | LED | P-I-N | 0.86 | 46.36 | 81 | 26.91 | Chem. Eng. J 454 (2023) 140284 |
| 2023 | 3000K | 500 | 138 | LED | N-I-P | 0.93 | 72 | 80.92 | 39.31 | Adv. Mater. 2024, 36, 2306870 |
| 2023 | 3000K | 200 | 56 | LED | N-I-P | 0.89 | 32 | 74.59 | 38.31 | Adv. Mater. 2024, 36, 2306870 |
| 2021 | 2700K | 500 | 169.8 | Warm-white LED | N-I-P | 0.92 | 83.15 | 77.26 | 34.64 | Adv. Mat. 33(27):2100770 |
| 2021 | 2700K | 200 | 71.1 | Warm-white LED | N-I-P | 0.88 | 37.74 | 71.07 | 33.27 | Adv. Mat. 33(27):2100770 |
| 2021 | 5000K | 200 | 60 | LED, BLD 1000 | N-I-P |  | 21.0 |  | 34.2 | Adv. Funct. Mater., 31 (2021), p. 2103614 |
| 2021 | - | 200 | 52.8 | LED, PBA-0822-CW-04A | N-I-P | 0.90 | 30.41 | 65.2 | 33.85 | Adv. Funct. Mater., 31 (2021), p. 2008908 |
| 2022 |  | 150 |  | LED | N-I-P | 0.9 | 106 | 78.89 | 20.63 | ACS Appl. Energy Mater. 2022, 5, 12, 14669–14679 |
| 2022 |  | 200 |  | Halogen | P-I-N | 0.86 | 106.89 | 79 | 33.68 | ACS Appl. Energy Mater. 2022, 5, 11, 13234–13242 |
| 2022 |  | 400 |  | Halogen | P-I-N | 0.88 | 205.15 | 78 | 33.95 | ACS Appl. Energy Mater. 2022, 5, 11, 13234–13242 |
| 2024 | 3000K | 400 | 120.8 | LED | P-I-N | 0.98 | 29.05 | 79.37 | 37.45 | Energy Environ. Sci., 2024,17, 1637-1644 |
| 2024 | 3000K | 200 | 60.4 | LED | P-I-N | 0.98 | 28.74 | 75.35 | 35.50 | Energy Environ. Sci., 2024,17, 1637-1644 |

**Table S15** Extracted charge (Q_ext_) and carrier mobility of perovskite solar cells with different buffer layers

| **Qext** | **Light intensity (mA/cm^2^)** | **BCP** | **Cu(BCP)(PEIE)** | **Carrier mobility (10^-3^cm^2^/Vs)** | **BCP** | **Cu(BCP)(PEIE)** |
| --- | --- | --- | --- | --- | --- | --- |
|  | 84.8 | 0.244 | 0.423 |  | 5.561 | 7.387 |
|  | 53.5 | 0.189 | 0.382 |  | 5.644 | 5.994 |
|  | 42.5 | 0.173 | 0.355 |  | 5.401 | 5.728 |
|  | 26.8 | 0.09 | 0.301 |  | 5.44 | 5.85 |
|  | 13.4 | 0.067 | 0.179 |  | 4.96 | 5.401 |
|  | 8.4 | 0.043 | 0.118 |  | 4.825 | 5.728 |
